# Multilevel genomics of colorectal cancers with microsatellite instability—clinical impact of *JAK1* mutations and consensus molecular subtype 1

**DOI:** 10.1186/s13073-017-0434-0

**Published:** 2017-05-24

**Authors:** Anita Sveen, Bjarne Johannessen, Torstein Tengs, Stine A. Danielsen, Ina A. Eilertsen, Guro E. Lind, Kaja C. G. Berg, Edward Leithe, Leonardo A. Meza-Zepeda, Enric Domingo, Ola Myklebost, David Kerr, Ian Tomlinson, Arild Nesbakken, Rolf I. Skotheim, Ragnhild A. Lothe

**Affiliations:** 1 Department of Molecular Oncology, Institute for Cancer Research, Oslo University Hospital, P.O. Box 4953, Nydalen, NO-0424 Oslo Norway; 2 K. G. Jebsen Colorectal Cancer Research Centre, Oslo University Hospital, P.O. Box 4953, Nydalen, NO-0424 Oslo Norway; 3 Norwegian Cancer Genomics Consortium, Oslo University Hospital, P.O. Box 4953, Nydalen, NO-0424 Oslo Norway; 4 Centre for Cancer Biomedicine, Institute for Clinical Medicine, University of Oslo, P.O. Box 4950, Nydalen, NO-0424 Oslo Norway; 5 Department of Tumor Biology, Institute for Cancer Research, Oslo University Hospital, P.O. Box 4953, Nydalen, NO-0424 Oslo Norway; 6 Genomics Core Facility, Department of Core Facilities, Institute for Cancer Research, Oslo University Hospital, P.O. Box 4953, Nydalen, NO-0424 Oslo Norway; 7 Wellcome Trust Centre for Human Genetics, University of Oxford, Roosevelt Drive, Oxford, OX3 7BN UK; 8 Department of Oncology, University of Oxford, Roosevelt Drive, Oxford, OX3 7DQ UK; 9 Department of Gastrointestinal Surgery, Oslo University Hospital, P.O. Box 4950, Nydalen, NO-0424 Oslo Norway

**Keywords:** Colorectal cancer, Consensus molecular subtypes, Immunogenicity, Immunotherapy resistance, *JAK1*, Microsatellite instability, Mutation, Neoantigen, Prognosis

## Abstract

**Background:**

Approximately 15% of primary colorectal cancers have DNA mismatch repair deficiency, causing a complex genome with thousands of small mutations—the microsatellite instability (MSI) phenotype. We investigated molecular heterogeneity and tumor immunogenicity in relation to clinical endpoints within this distinct subtype of colorectal cancers.

**Methods:**

A total of 333 primary MSI+ colorectal tumors from multiple cohorts were analyzed by multilevel genomics and computational modeling—including mutation profiling, clonality modeling, and neoantigen prediction in a subset of the tumors, as well as gene expression profiling for consensus molecular subtypes (CMS) and immune cell infiltration.

**Results:**

Novel, frequent frameshift mutations in four cancer-critical genes were identified by deep exome sequencing, including in *CRTC1*, *BCL9*, *JAK1*, and *PTCH1. JAK1* loss-of-function mutations were validated with an overall frequency of 20% in Norwegian and British patients, and mutated tumors had up-regulation of transcriptional signatures associated with resistance to anti-PD-1 treatment. Clonality analyses revealed a high level of intra-tumor heterogeneity; however, this was not associated with disease progression. Among the MSI+ tumors, the total mutation load correlated with the number of predicted neoantigens (*P* = 4 × 10^−5^), but not with immune cell infiltration—this was dependent on the CMS class; MSI+ tumors in CMS1 were highly immunogenic compared to MSI+ tumors in CMS2-4. Both *JAK1* mutations and CMS1 were favorable prognostic factors (hazard ratios 0.2 [0.05–0.9] and 0.4 [0.2–0.9], respectively, *P* = 0.03 and 0.02).

**Conclusions:**

Multilevel genomic analyses of MSI+ colorectal cancer revealed molecular heterogeneity with clinical relevance, including tumor immunogenicity and a favorable patient outcome associated with *JAK1* mutations and the transcriptomic subgroup CMS1, emphasizing the potential for prognostic stratification of this clinically important subtype.

See related research highlight by Samstein and Chan 10.1186/s13073-017-0438-9

**Electronic supplementary material:**

The online version of this article (doi:10.1186/s13073-017-0434-0) contains supplementary material, which is available to authorized users.

## Background

Colorectal cancers (CRCs) with the microsatellite instability (MSI) phenotype are defined by high rates of insertions and deletions (indels) of nucleotides in short, repetitive sequences [1–3]. This is caused by a defective DNA mismatch repair (MMR) machinery, either by epigenetic silencing, primarily of *MLH1* [4–6], or through somatic or germ line mutations [7–11]. MSI occurs in a wide range of cancer types but is prevalent in only a few [12–15]. In CRC, the MSI+ subgroup accounts for approximately 15% of cases and is characterized by a low level of DNA copy number aberrations but a higher frequency of small mutations (single nucleotide variants (SNVs), and indels) than most other types of cancer [16, 17]. The mutation profiles of MSI+ CRCs differ from those of microsatellite stable (MSS) CRCs, for example, by a strong enrichment for *BRAF* mutations [18]. Furthermore, low complexity sequences with short tandem repeats are prone to indel mutations and are found in the coding regions of several cancer-critical genes [19–21], including *TGFBR2*, which was the first MSI target gene to be discovered [22–24]. However, large-scale analysis of indel mutations has been challenging [25, 26], and a recent paper describing frameshift mutations in *RNF43* in 80% of MSI+ CRCs clearly demonstrates that even highly prevalent indel mutations have gone unnoticed [27].

CRC has a high world-wide incidence and mortality rate [28], but compared with the MSS subgroup, patients with MSI+ tumors have a favorable prognosis in the primary setting [3, 29, 30]. This is possibly explained by tumor immunogenicity and the high level of lymphocyte infiltration [31, 32]. Immunogenicity in MSI+ tumors may be a result of the high mutation load and subsequent expression of mutated, tumor-specific peptides as neoantigens [33]. Neoantigens are presented on the tumor cell surface by class I HLA molecules and have the potential to activate cytotoxic T cells. Expression of neoantigens, computationally predicted based on mutation profiling, has been shown to be associated with a favorable patient outcome across tumor types [34]. In CRC, there are strong indications that the number of predicted neoantigens is also prognostic [35–37], but the subgroup of MSI+ tumors has not been specifically analyzed in this context. In the metastatic setting, MSI+ tumors are associated with a poor prognosis [38], but patients generally respond well to immunotherapy by anti-PD-1 immune checkpoint inhibition [39]. However, disease control is not obtained in all cases, and prediction of treatment response is currently a research area of high interest [40, 41].

At the gene expression level, an international consortium has recently described four consensus molecular subtypes (CMS) of CRC [42]. The majority of MSI+ tumors are found in CMS1, which is a subtype characterized by a high mutation load and infiltration of immune cells in the tumor microenvironment [42, 43]. CMS classification has clinical value independent of cancer stage, and patients with mesenchymal CMS4 tumors have a poor outcome, while CMS1 is associated with a poor patient survival after relapse. The heterogeneity and clinical value of CMS classification specifically within the subgroup of MSI+ tumors remains unknown.

In the present study, we have performed deep exome sequencing of MSI+ CRCs, integrated with DNA copy number and gene expression analyses, to (i) identify potential novel mutations and (ii) analyze the mutation-associated immunogenicity of the tumors, in relation to both CMS and clinical endpoints.

## Methods

### Patient samples

**Fig. 1:**
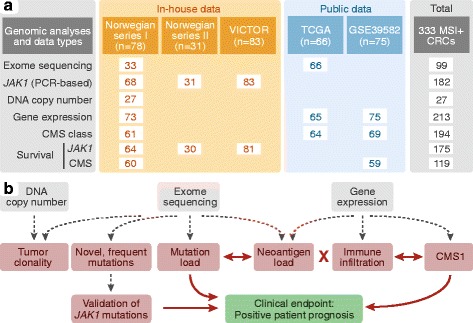
Patient samples and data analyses. **a** A total of 333 MSI+ CRCs were analyzed, including 192 in-house tumor samples and publicly available data from 141 patients; 248 were analyzed for gene mutations and 213 for gene expression. Among the 68 samples in Norwegian series I analyzed for *JAK1* mutations by PCR-based analysis, 33 were exome sequenced, 27 were analyzed for DNA copy number aberrations, and 63 were analyzed for gene expression. **b** Molecular result parameters are shown in *red*, clinical endpoints in *green*, and the data types used for analysis in *grey*. Significant associations are indicated by *red arrows* and the *red cross* indicates no association

A total of 333 primary MSI+ CRCs from five patient series were analyzed for mutations, DNA copy number, and/or gene expression (Fig. 1a). This includes tumors from two Norwegian series (*n* = 78 and 31) and British patients recruited to the VICTOR adjuvant randomized trial (*n* = 83, 81 with clinical annotation available) [44], as well as publicly available data from The Cancer Genome Atlas (TCGA; *n* = 66) [16] and a French multicenter cohort (*n* = 75; Gene Expression Omnibus (GEO), accession number GSE39582) [45].

Fresh-frozen tumor specimens and matched normal colonic mucosa have consecutively been collected from patients treated for primary CRC at Oslo University Hospital, Norway since 2005. This patient series is referred to as Norwegian series I and all 78 patients treated for MSI+ CRC before May 2013 were included. For validation of *JAK1* mutation prevalence, another 31 MSI+ tumors from an anonymized Norwegian CRC patient series treated surgically at hospitals in the Oslo region from 1987 to 1989 were analyzed (Norwegian series II; Table 1; Fig. 1a).

**Table 1:** Clinicopathological data for MSI+ CRCs analyzed in-lab

Clinicopathological parameters	Norwegian series I (*n* = 78)		Norwegian series II (*n* = 31)	VICTOR (*n* = 81)
	All patients	Exome sequenced (*n* = 33)		
Patient age at diagnosis (years)				
Mean	75	74	66	65
Min	37	37	26	38
Max	93	93	92	89
Patient gender				
Female	55	23	18	45
Male	23	10	13	36
Tumor localization				
Right	68	31	20	66
Left	6	1	7	13
Rectum	4	1	4	-
Unknown	-	-	-	2
Tumor stage				
I	16	1	3	-
II	42	23	17	54
III	15	9	8	27
IV	5	-	3	-
5-year survival rates				
Overall	71%	85%	83%	82%
Relapse-free	70%	82%	72%	NA

*NA* not available

DNA was extracted using a standard phenol/chloroform protocol or a magnetic bead approach according to the manufacturer’s recommendations (Maxwell 16 DNA Purification Kit; Promega, Madison, WI, USA). Microsatellite instability status of the tumors was determined using the consensus markers provided by the National Cancer Institute (Bethesda marker panel), as previously described [46]. The DNA promoter methylation status of *MLH1* was analyzed by quantitative methylation-specific PCR, as previously described [47]. Primers and probe (Additional file 1: Table S1) were designed using the Methyl Primer Express 1.0 and the Primer Express 3.0 software (Thermo Fisher Scientific Inc., Waltham, MA, USA). RNA was extracted using the Qiagen Allprep DNA/RNA/miRNA Universal kit, according to the manufacturer’s instructions (Qiagen, GmBH, Hilden, Germany).

### Exome sequencing and mutation analysis

Matched tumor and normal colonic mucosa samples from 33 patients in Norwegian series I were exome sequenced (Table 1; Fig. 1a). Exome libraries were generated from 1 μg of genomic DNA using the Agilent SureSelectXT Human All Exon V5 kit (Agilent, Santa Clara, CA, USA), and 2 × 100-base-pair paired-end sequencing was performed using the Illumina HiSeq 2500 system and sequencing by synthesis chemistry (Illumina, San Diego, CA, USA) at the Oslo University Hospital Genomics Core Facility (The Norwegian Radium Hospital, Oslo, Norway). We received the sequencing reads and performed sequence alignment using BWA version 0.7.8 [48]. Sequence Alignment Map (SAM) files were converted to Binary Alignment Map (BAM) files using Picard version 1.102 (http://broadinstitute.github.io/picard/). SAMtools version 0.1.19 (http://samtools.sourceforge.net/) was used for sorting and indexing. The Picard toolbox was applied to remove duplicates. Subsequent refinements of the BAM files were done according to The Genome Analysis Toolkit Best Practices guidelines (https://www.broadinstitute.org/gatk/guide/best-practices). Potential indel regions were realigned using IndelRealigner and the quality of the alignment was recalibrated using BaseRecalibrator and AnalyzeCovariates. All SNVs were called using MuTect v.1.1.7 [49] and indels were scored using the program Strelka v.1.0.14 [50]. The sequence variants were annotated using ANNOVAR [51] and the Ensembl Variant Effect Predictor v.79. The coverage threshold for each candidate somatic mutation was set to minimum 10× for normal tissue and 15× for tumor samples, and loci where <5% of the reads supported a mutation were filtered out. A single mutated read from the normal tissue was accepted to allow for minute sample contamination or the presence of circulating tumor cells. All analyses were done using human genome build hg19.

Significance of candidate mutations was evaluated using MutSigCV v.1.4 [52], and genes with a false discovery rate (*q* value) below 0.05 were considered significantly mutated above the background mutation rate. For comparison, the Cancer Gene Census (CGC) v.73 was downloaded from the COSMIC database (http://cancer.sanger.ac.uk/census), and the 571 listed genes are referred to as cancer-critical genes.

Candidate genes with novel frequent indels were additionally screened for hotspot indels in homopolymeric regions using the SAMtools (v.0.1.19) mpileup command. For in silico validation, aligned exome sequencing data from 78 MSI+ CRCs in TCGA were downloaded from the Cancer Genomics Hub. Using a threshold of a minimum of seven reads at each homopolymer with a mutation hotspot, 66 tumors were retained for mutation analyses using the SAMtools mpileup command. The mutation load (total number of mutations) was determined for 60 of the tumors using the same analysis pipeline as for the in-house exome sequencing data.

### Estimation of MSI level

The level of MSI per sample was estimated from the exome sequencing data using the algorithm MSIsensor [53] with default settings. For a catalogue of microsatellites in the reference genome (homopolymers of a minimum of 5 base pairs and microsatellites with maximum repeat unit length 5), the number of loci with sufficient sequencing coverage to be scored per sample (minimum 20 reads) was detected by mapping sequencing reads from BAM files. Unstable microsatellites were detected by counting mapped reads with non-reference repeat lengths, and somatic events were detected as sites with significantly different mutant read count distributions between paired tumor–normal samples. An MSI score was calculated per tumor, representing the percentage of unstable microsatellites (microsatellites with indels). The MSI score was not associated with the number of microsatellites scored per sample (Spearman correlation −0.03, *P* = 0.8).

### Mutation signatures

The mutation signatures designated in COSMIC [54] were analyzed per tumor based on SNVs and their sequence context, considering the immediately flanking 5′ and 3′ nucleotides using the R package SomaticSignatures [55] with default settings.

### PCR-based validation of *JAK1* indels in independent patient series

Frameshift mutations in four hotspots in homopolymeric regions of *JAK1* were analyzed by PCR-based fragment analyses in a total of 182 MSI+ CRCs (Fig. 1a). Fluorescently labeled primers were designed using Primer3 [56] (Additional file 1: Table S1). The four *JAK1* fragments were amplified in multiplex PCR using the Qiagen Multiplex PCR Kit according to the manufacturer’s recommendations. One primer per fragment was labeled with a fluorescent dye from the G5 dye set, and GS500 LIZ was used as a size standard (Thermo Fisher Scientific Inc.). All fragments were run in duplicate on an ABI 3730 DNA Analyzer, with default Microsatellite Analysis settings, and analyzed by the GeneMapper software v.3.7 (Thermo Fisher Scientific Inc.). DNA from disease-free individuals and water served as normal and negative controls, respectively. Electropherograms were scored independently by two researchers.

### High resolution DNA copy number analysis

DNA copy number data were generated for 27 of the 33 exome sequenced MSI+ tumors in Norwegian series I using Affymetrix Genome-Wide Human SNP Arrays 6.0 (Affymetrix Inc., Santa Clara, CA; Fig. 1a). DNA (1 μg) was prepared according to the Affymetrix SNP6.0 Cytogenetics Copy Number Assay (revision 3) and hybridized onto microarrays according to the Affymetrix Genome-Wide Human SNP Nsp/Sty 6.0 User Guide (revision 4). The raw copy number data were preprocessed according to the PennCNV protocol [57] adapted for Affymetrix genotyping arrays (http://www.openbioinformatics.org/penncnv/penncnv_tutorial_affy_gw6.html) using HapMap samples [58] as reference. Single-sample segmentation and calculation of allele-specific and absolute DNA copy numbers were performed using the algorithm ASCAT (v.2.3) [59], with the penalty constant for segmentation by the Allele-Specific Piecewise Constant Fitting algorithm set to 50. The sex chromosomes were excluded from further analyses.

For comparison of DNA copy number loss and gain across samples with different segment breakpoint positions, an artificial splitting of the data into smaller segments including all breakpoint positions across the sample set was performed. For each of these smallest regions of overlap, the copy number value of the original, larger segment was kept. Copy numbers per segment were adjusted according to the overall ploidy of each tumor. Regions with loss of heterozygosity (LOH) were identified as segments with DNA copy number equal to zero for one allele and higher than one for the other allele. Copy number neutral LOH was defined as DNA copy number equal to zero for one allele and equal to the overall ploidy of the sample for the other allele. The proportion of the genome affected by copy number aberrations (CNAs) was estimated for all samples as the percentage of base pairs with a total copy number different from the overall ploidy of the sample.

### Modeling of tumor clonality

Tumor clonality was modeled for the 27 MSI+ tumors in Norwegian series I analyzed by both exome sequencing and DNA copy number variation using the R package SciClone with default settings (except the threshold for the minimum total read count set to 50) [60]. The variant allele frequency (VAF) of each SNV and indel, defined as the proportion (percentage) of reads of the variant allele compared with the total number of reads at the respective locus, was used as input, and SciClone identified sample-wise mutation clusters (representing genetically distinct cellular populations/subclones) based on SNVs and indels unaffected by CNAs and LOH using a variational Bayesian mixture model. The mutation cluster with a mean VAF of approximately 50% of the aberrant cell fraction (estimated using ASCAT) corresponds to an early clone with pervasive heterozygous mutations, and mutations in this clone were scored as truncal. Mutations in clones with lower and higher mean VAFs were scored as subclonal and homozygous, respectively. The number of clones per tumor was summarized from distinct mutation clusters of truncal and subclonal mutations.

For comparison, tumor clonality was also modeled using the R package EXPANDS [61], with maxScore set to 1.5 and precision to 0.02. Predicted subclones containing less than 5% of the mutations per tumor were not considered. EXPANDS models the cellular prevalence of each SNV as a probability distribution based on its VAF, adjusted for the DNA copy number at the mutated locus.

### Gene expression analyses

Genome-wide exon-level expression analysis was done for a total of 73 MSI+ tumors in Norwegian series I, including 63 of the 68 tumors analyzed for *JAK1* mutations, using Affymetrix GeneChip Human Exon 1.0 ST and Human Transcriptome 2.0 Arrays (Affymetrix Inc., Santa Clara, CA, USA). These data have partly previously been published (*n* = 39 samples; GSE24550, GSE29638, and GSE69182), and the remaining samples (*n* = 34) have been deposited to the NCBI’s GEO with accession number GSE79959. Raw intensity data were background corrected, quantile normalized, and summarized at the gene-level according to the robust multi-array average (RMA) approach implemented in the Affymetrix Expression Console 1.1 software. For samples analyzed on the Affymetrix Human Transcriptome 2.0 Array, the modified Signal Space Transformation algorithm of RMA was used. Gene expression data generated from the two different platforms were matched by HUGO gene symbols and merged by batch correction using ComBat [62] implemented in the R package SVA.

Classification of the tumors according to CMS was performed using the random forest predictor implemented in the R library CMSclassifier [42]. A default posterior probability of 0.5 was used as a threshold for sample classification, assigning a CMS class to 61 (84%) of the 73 tumors.

In addition, gene expression data and CMS assignments for 65 and 75 MSI+ tumors in TCGA and GSE39582 [45], analyzed by RNA sequencing and Affymetrix HG U133 Plus 2.0 arrays, respectively, were downloaded from the Colorectal Cancer Subtyping Consortium web site at SAGE Synapse (https://www.synapse.org/#!Synapse:syn2623706/wiki/67246). Of these tumors, 64 and 69 were assigned to a CMS class, respectively. For GSE39582, clinical data (reporting relapse-free survival (RFS) and overall survival (OS) for 67 of the CMS classified patients) were obtained via the GEO accession number (Additional file 1: Table S2). Patients (*n* = 8 with CMS classification) reported to have an event for OS and simultaneously censored for RFS were excluded from survival analyses, due to non-compliance with the definition of end-points used in this study [63].

Gene set expression enrichment analyses were performed using the R package GSA [64] and a customized collection of 51 gene sets relevant to CRC (Additional file 1: Table S11). Sample-wise gene set expression enrichment scores were calculated using the R package GSVA [65].

Tumor immunogenicity, evaluated based on gene expression, was analyzed by the “immunophenoscore”, using the published R code [41]. For marker genes with missing expression values (7 of the 162 genes on Human Exon 1.0 arrays), imputation was done based on the overall mean expression per sample, using the impute.knn function in the R package impute. The level of infiltration of immune cells in the tumors was also evaluated based on gene expression using the R packages ESTIMATE [66] and MCPcounter v.1.1 [43]. For comparison with the MSI+ tumors, gene expression data from 160 MSS tumors from Norwegian series I, included in the previously published GEO records, were also analyzed for immune cell infiltration.

### Neoantigen prediction

Mutation-associated immunogenicity was analyzed from exome sequencing and gene expression data by computational prediction of neopeptides able to bind to patient-specific HLA molecules. Genotyping of the individual patients for the HLA class I genes *HLA-A*, *B*, and *C* at four-digit resolution (Additional file 1: Table S3) was done from merged sequencing BAM files of paired tumor–normal samples for each patient using the algorithm Polysolver with default settings (previously reported mean overall accuracy 97%) [67]. Prior to neopeptide prediction, the list of mutations (Additional file 1: Table S4) was filtered sample-wise based on gene expression, and only mutations in genes with an expression level above the median level per tumor were kept, retaining a median of 47% of the mutations per tumor (range 42 to 54%). All possible peptides of length 8–11 amino acids containing these mutations were predicted using Topiary 0.0.14 (https://pypi.python.org/pypi/topiary), with prediction of binding affinities for the patient-specific HLA alleles using NetMHCpan 2.8 [68]. Only peptides with IC_50_ below 500 and a percentile rank of predicted IC_50_ values across interactions between all peptides and each HLA allele per tumor below 2 were retained. The resulting predicted neoantigens were further filtered to exclude peptides encoded from other, non-mutated loci in the human genome by searching for perfect matches of the peptide sequences in UniProtKB release 2015_11 using the web application Peptide Match service for UniProt Knowledgebase [69]. Lastly, to include only peptides predicted to be generated by cleavage in proteasomes, the list of neoantigens was filtered to include only peptides with predicted C-terminal cleavage sites using the stand-alone software package of NetChop 3.1 [70]. When summarizing the number of neoantigens per tumor, peptides predicted to bind to several HLA alleles were counted only once.

### Survival analyses

Univariable and multivariable survival analyses were conducted with Cox’s proportional hazards regression, with calculation of *P* values from Wald’s tests for predictive potential using the SPSS software v.21 (IBM Corporation, Armonk, NY, USA). Kaplan–Meier survival curves were compared with the log-rank test. Five-year RFS (considering relapse or death from any cause as events, and censoring in the case of no event within five years) and OS (time to death from any cause) were used as endpoints, according to the definition by Punt et al. [63]. Time to event or censoring was calculated from the time of surgery.

## Results

### Molecular overview of MMR deficiency in MSI+ CRC

A total of 333 MSI+ CRCs were analyzed in the study; an overview of the genomic analyses and results is shown in Fig. 1 (parts a and b, respectively).

**Fig. 2:**
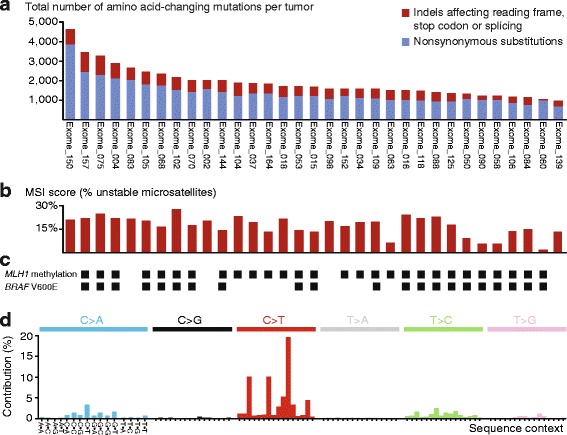
Somatic exonic mutation profiles of 33 MSI+ CRCs. **a** The total number of amino acid-changing mutations per tumor ranged from 957 to 4614 (median 1676). The tumors are sorted by mutation load. **b** The MSI score, calculated as the percentage of somatically unstable microsatellites in the exome of each tumor (microsatellites with indels), was more strongly associated with the number of indels than substitutions (Spearman correlations 0.7 and 0.4, respectively). **c** *MLH1* promoter hypermethylation and *BRAF V600E* mutations were found in 28 (85%) and 21 (64%) of the tumors, respectively. There was no difference in the MSI score between tumors with and without *MLH1* methylation. **d** Collection across all 33 tumors of substitutions in each of six categories (indicated in the *top panel*), classified according to sequence context (flanking nucleotides are indicated on the *horizontal axis*). The mutation distribution corresponds to mutation signature 6, as designated in COSMIC, which is associated with defective MMR

Tissue samples from 33 stage I–III MSI+ CRCs and matched normal colonic mucosa in Norwegian series I (Table 1) were subjected to exome sequencing with an average depth of 273× and 109×, respectively. A total of 83,706 somatic small exonic mutations, comprising 65,880 SNVs and 17,826 indels, passed the quality filtering (Additional file 1: Tables S4 and S5). The number of amino acid-affecting mutations per tumor ranged from 957 to 4614 (median 1676; Fig. 2a), and the ratio of amino acid-affecting mutations to synonymous SNVs and in-frame indels was relatively consistent among the tumors (average 2.94, standard deviation 0.25; Additional file 1: Table S5). An MSI score representing the percentage of somatically unstable microsatellites per tumor was calculated using the algorithm MSIsensor (Fig. 2b; Additional file 1: Table S5). The MSI score was strongly correlated with the number of indels per tumor, as expected, but only weakly associated with the number of SNVs (Spearman correlations 0.7 and 0.4, respectively, *P* = 5 × 10^−6^ and 0.01; Additional file 2: Figure S1a).

Consistent with their MSI phenotype, 28 (85%) of the tumors had methylation of the *MLH1* promoter (Fig. 2c). Compared to tumors without methylation (*n* = 5), this was associated with a significant down-regulation of *MLH1* gene expression (*P* = 0.02 by Welch’s *t*-test; Additional file 2: Figure S1b). The five tumors without *MLH1* promoter methylation had down-regulated expression of seven other known MMR genes (Additional file 2: Figure S1c), and the sample-wise gene set enrichment score for these seven genes (calculated using the R package GSVA) was significantly lower in the unmethylated compared to the methylated samples (mean difference −0.5, *P* = 0.05). There was no difference in the MSI score between tumors with and without *MLH1* promoter methylation (mean MSI scores 17 and 19%, respectively, *P* = 0.2); however, lack of methylation was associated with a younger age at diagnosis (mean age 55 years compared to 77 for patients with methylation, *P* = 0.01). Accordingly, familial predisposition cannot be excluded, in particular for the two youngest patients (aged 37 and 48 years).

The most common single nucleotide substitution type was C > T/G > A transitions. Analyzing all substitutions and their sequence context (the two flanking nucleotides) across all 33 MSI+ tumors, the distribution of mutations was found to correspond to mutation signature 6, as designated in COSMIC [54] (Fig. 2d). This signature is associated with hypermutation caused by MMR deficiency and was the predominant mutation signature also in all the tumors individually (Additional file 2: Figure S1d). Although ten (30%) of the tumors had amino acid-changing mutations in *POLE* (Additional file 1: Table S4), none of these were in known pathogenic hotspots [71], and none of the tumors had a mutation signature indicative of *POLE*-associated hypermutation.

In contrast to SNVs and indels, the tumors displayed few CNAs and the median proportion of the genome affected by CNAs was 2% (Additional file 2: Figures S1e and f; Additional file 1: Table S6).

### Novel frequent frameshift indels in cancer-critical genes

In the 33 exome sequenced tumors, a total of 228 genes were identified as significantly mutated by MutSigCV (*q* values <0.05; Additional file 1: Table S7), including *PHACTR4*, *RPL22*, *TFAM*, *TMBIM4*, *TTK, VPS37B*, and *WASF3* (ranked by *q* values). Compared to the whole exome, there was a clear enrichment for indels over SNVs in the significantly mutated genes; the average proportion of mutations that were indels was 80% among the tumors, significantly larger than the average of 27% for the full exome (*P* = 8 × 10^−33^, paired *t*-test; Additional file 2: Figure S1g). Also among the 571 genes included in the CGC, a modest increase in the sample-wise ratio of indels to SNVs was observed (the average ratio across the tumors was 32%), compared to the whole exome (*P* = 1 × 10^−6^).

**Fig. 3:**
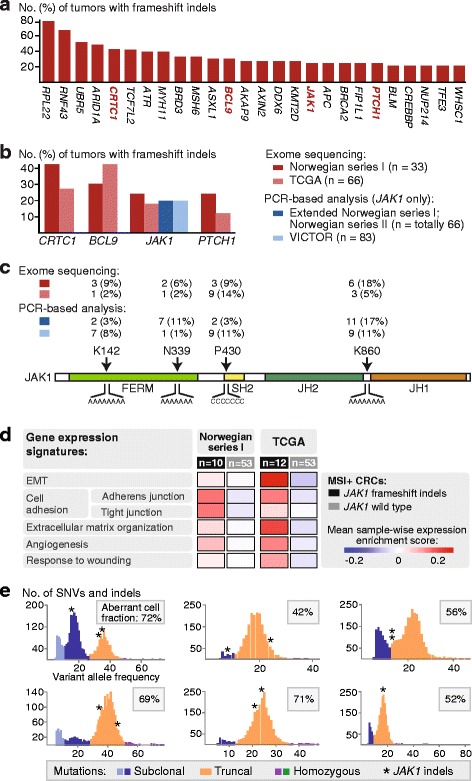
Cancer-critical genes with frequent frameshift mutations. **a** Mutation frequency of cancer-critical genes with most frequent indels affecting the reading frame, splice sites, or stop codons. *CRTC1* (mutation frequency 42%), *BCL9* (30%), *JAK1* (24%), and *PTCH1* (24%) were identified as novel frequently mutated genes. **b** Mutation validation analyses in MSI+ CRCs in independent patient series. **c** The *JAK1* frameshift indels were found in four hotspot amino acid positions (GenBank accession NP_002218; 1154 amino acids). The number of mutations detected in each patient series and mutation hotspot is shown, confirming frequent mutations upstream of the *JAK1* kinase domain (JH1). The patient series analyzed are indicated (*top left*) using the same color code as in **b**. All four homopolymers of length ≥6 in *JAK1* are indicated below the protein. Protein domains are indicated by color; *SH2* Src Homology 2, *FERM* (F, 4.1 protein; E, ezrin; R, radixin; M, moesin), *JH1* kinase domain, *JH2* pseudokinase domain. **d** Gene signatures of five processes previously found to be associated with innate resistance to anti-PD-1 treatment in melanomas were over-expressed in MSI+ CRCs with versus without *JAK1* mutations in both Norwegian series I and TCGA. **e** Six of the tumors in Norwegian series I with *JAK1* indels were available for clonality analyses. All these tumors had at least one *JAK1* indel (*asterisk*) scored as truncal, and all *JAK1* mutations were heterozygous. Each plot represents one sample and separate tumor clones, identified as mutation clusters with different variant allele frequencies, are indicated with different colors

Among genes in the CGC, frequent frameshift indels were detected in several genes not previously reported to be highly mutated in CRC, including *CRTC1* (mutation frequency 42%), *BCL9* (30%), and *PTCH1* (24%) (Fig. 3a). In addition, 15 of the tumors (45%) were found to have frameshift mutations in members of the JAK-STAT pathway (Additional file 1: Table S8). The most commonly mutated gene was *JAK1*, in which 24% of the tumors (8 of 33) had one or more single base pair frameshift indels (Fig. 3a). For *CRTC1* and *BCL9*, tumors with mutations had significantly higher MSI scores than the respective wild-type tumors (on average 4% more unstable microsatellites; *P* < 0.04 for both genes by Welch’s *t*-test); however, there was no significant difference in the MSI score between tumors mutated and wild type for *PTCH1* or *JAK1* (*P* > 0.3), indicating that these mutations are not associated with the level of tumor hypermutation. None of the mutations were associated with *MLH1* promoter methylation (by Fisher’s exact test). For validation, we searched for the novel frequent frameshift mutations in publicly available exome sequencing data from 66 additional MSI+ CRCs from TCGA. The mutation frequencies of *CRTC1*, *BCL9*, *JAK1*, and *PTCH1* were 27, 42, 18, and 12%, respectively (Fig. 3b; Additional file 1: Table S8).

### Validation and transcriptional consequences of *JAK1* loss-of-function mutations

Because of the recently suggested predictive value of *JAK1* mutations indicating resistance to anti-PD-1 treatment [72, 73], this gene was selected for mutation validation analyses by PCR-based fragment analyses. Frameshift mutations in the four hotspots identified by exome sequencing were verified in the 33 tumors. These hotspots were all in homopolymers upstream of the *JAK1* kinase domain (Fig. 3c), and the mutations have previously been described as truncating, loss-of-function mutations [74]. For an additional 149 MSI+ CRCs, *JAK1* mutation frequency was 20%, both in independent Norwegian patients (mutations in 13 of 66 tumors) and British patients from the VICTOR trial (17 of 83) (Fig. 3b; Additional file 1: Table S8). In summary, the mutation frequency of *JAK1* across all 248 primary MSI+ CRCs analyzed was 20%.

*JAK1* gene expression was down-regulated in MSI+ tumors in Norwegian series I with versus without *JAK1* mutations (Additional file 2: Figure S2a). Gene set expression enrichment analysis also revealed a modest depletion of JAK-STAT signaling in the mutated tumors (Additional file 1: Table S9). Resistance to anti-PD-1 treatment in hypermutated tumors with *JAK1* loss-of-function mutations has been proposed to be linked to reduced expression of the PD-L1 ligand, mediated by reduced signaling in the interferon gamma (IFN-γ) receptor pathway [73]. In Norwegian series I, the sample-wise enrichment scores of a six-gene IFN-γ expression signature [75] (calculated using the R package GSVA) was significantly lower in *JAK1* mutated compared to wild-type tumors (Additional file 2: Figure S2b). Also, both PD-1 signaling and gene expression of the PD-L1 ligand (*CD274*) was significantly down-regulated in the mutated tumors (Additional file 2: Figure S2c, d). To further explore a potential association with immune evasion, tumors were analyzed for the gene expression-based immunophenoscore [41]. Tumors with *JAK1* mutations had a lower score than wild-type tumors specifically for the antigen processing component (MHC score, mean difference −0.3, *P* = 0.01 by Welch’s *t*-test), indicating immune evasion, similarly to what has been observed in endometrial cancer [76]. The total immunophenoscore (IPS score) was also lower in the mutated tumors (mean difference −0.8, *P* = 0.08), predicting a poor response to immune checkpoint inhibition [41]. In concordance, tumors with mutations were also found to be positively enriched for transcriptional signatures of biological processes described to be up-regulated in melanomas with innate resistance to anti-PD-1 treatment [40]. These processes include epithelial-to-mesenchymal transition (EMT), cell adhesion, extracellular matrix organization, angiogenesis, and response to wounding; sample-wise enrichment scores for gene signatures of all these five processes were higher in tumors with *JAK1* mutations in both Norwegian series I and TCGA (Fig. 3d; Additional file 1: Table S9).

### Clonality of MSI+ CRCs and *JAK1* mutations

Resistance to anti-PD-1 treatment is found in tumors with homozygous, truncating *JAK1* mutations (mutation accompanied by LOH at the *JAK1* locus), resulting in complete loss of protein function [73]. To analyze the zygosity of mutations, we did computational modeling of tumor clonality based on the VAFs of exonic SNVs and indels unaffected by CNAs in the 27 tumors with both data types available, using the algorithm SciClone [60] (Additional file 2: Figure S3a, b; Additional file 1: Table S10). All tumors except three (89%) revealed intra-tumor heterogeneity with more than one subclone, and the number of clones per tumor was independent of the number of mutations (Additional file 2: Figure S3c). The pattern of clonality was similar among the tumors and the majority of mutations were found to be heterozygous and truncal (i.e*.*, pervasive in all cancer cells in the tumor) in all tumors except one (96% of tumors; Additional file 2: Figure S3d). The proportion of truncal mutations in the full exome and genes in the CGC was proportional across the tumors, with a median of 85 and 82%, respectively (Additional file 2: Figure S2e). For comparison, tumor clonality was also modeled based on the algorithm EXPANDS [61], supporting the prediction of multiple subclones in the majority of tumors (all except one; details in Additional file 2: Supplementary text).

All truncating *JAK1* indels were heterozygous and unaffected by LOH (Fig. 3e), but all mutated tumors had at least one truncal *JAK1* mutation. Considering the other genes with novel frequent frameshift mutations, *BCL9* and *PTCH1* mutations were also mostly heterozygous and truncal, although one tumor had a homozygous *BCL9* mutation and both genes were found to have a subclonal mutation in one tumor each. In contrast, mutations in *CRTC1* were frequently subclonal and considering both indels and SNVs, *CRTC1* was indeed the cancer-critical gene with most frequent subclonal mutations (in 30% of the 27 tumors).

### The mutation load of MSI+ CRCs is correlated with the predicted neoantigen load, but not with immune cell infiltration

**Fig. 4:**
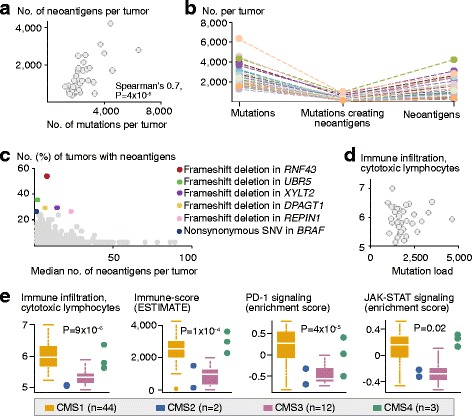
Predicted neoantigen load correlates with mutation load, while immune infiltration is highest in CMS1. **a** Among the 33 exome-sequenced tumors, there was a strong correlation between the number of exonic mutations (including synonymous) and predicted neoantigens. **b** There was not a one-to-one correspondence among the number of mutations (*left*), mutations predicted to create neoantigens (*middle*), and predicted neoantigens (*right*) per tumor (each tumor is indicated with a separate color), showing that individual mutations generate several neoantigens per tumor. **c** The individual mutations (represented by a *dot*) predicted to create most neoantigens per tumor (*horizontal axis*) were typically not recurrent in a large proportion of the tumors (*vertical axis*). However, several mutations (indicated by colors) created neoantigens in several tumors. **d** The number of amino acid changing mutations per tumor (*horizontal axis*) was not associated with the level of infiltration of cytotoxic lymphocytes (Spearman’s correlation −0.06, *P* = 0.8). **e** In contrast, among all MSI+ tumors in Norwegian series I, infiltration of cytotoxic lymphocytes and the ESTIMATE immune-score, as well as PD-1 and JAK-STAT signaling (based on gene expression) were significantly higher in CMS1 compared to CMS2-4 (Welch’s *t*-test; although high also in CMS4)

To analyze the association between genetic changes and infiltration of immune cells, the latter was evaluated based on gene expression using the algorithm MCPcounter [43] and the immune-score ESTIMATE [66]. In agreement with their high mutation load, the MSI+ tumors in Norwegian series I (*n* = 73) had a higher level of infiltration of cytotoxic lymphocytes and immune-score compared to the MSS tumors (*n* = 160; Additional file 1: Figure S4a). In the exome sequenced MSI+ tumors, neoantigens with high binding affinities for the patient-specific class I HLA molecules were predicted from small exonic mutations in highly expressed genes (expression level above the median across all genes per tumor). The number of predicted neoantigens per tumor (median 1169; range 376 to 4245) was correlated to the number of exonic mutations (also including synonymous; Spearman’s correlation 0.7, *P* = 4 × 10^−5^; Fig. 4a). However, this relationship was not one-to-one, indicating generation of multiple neoantigens from individual mutations (Fig. 4b). As expected, all mutations creating the largest numbers of neoantigens were frameshift indels (Additional file 2: Figure S4b), and the mean ratios between the number of neoantigens and mutations per tumor were 1.3 and 0.5 for frameshift mutations and SNVs, respectively (*P* = 4 × 10^−10^ by paired samples *t*-test). Still, the numbers of mutations were correlated to the corresponding numbers of neoantigens for both frameshift mutations and nonsynonymous SNVs separately (Spearman’s correlation 0.6; *P* = 2 × 10^−4^ and *P* = 1 × 10^−4^, respectively; Additional file 2: Figure S4c). Considering also the clonality of the mutations, a strong correlation (Spearman 0.9; *P* = 7 × 10^−12^) between the number of neoantigens predicted from truncal mutations and the total number of mutations per tumor was found.

Mutations predicted to be highly immunogenic were mostly non-recurrent among the tumors (Fig. 4c). Altogether, only 0.5 and 14.7% of the SNVs and frameshift mutations, respectively, were predicted to create neoantigens in more than one tumor. Mutations in *RNF43*, *UBR5*, *XYLT2*, *DPAGT1*, *REPIN1*, and *BRAF* (*V600E*) were most frequently immunogenic (in 55, 36, 30, 30, 27, and 27% of the 33 tumors, respectively).

Surprisingly, there were no associations between the mutation load (or predicted neoantigen load) and the level of immune cell infiltration or the immunophenoscore among the MSI+ tumors in neither Norwegian series I (Fig. 4d; Additional file 2: Figure S4d) nor TCGA. Similarly, no indication of increased immune evasion, analyzed as PD-1 signaling, was detected in tumors with a high mutation load (Additional file 2: Figure S4e).

There was no difference in the mutation load (or predicted neoantigen load) between tumors with and without *JAK1* frameshift indels (*P* = 0.5; Additional file 2: Figure S4f). However, in concordance with the observed down-regulation of PD-1 signaling associated with *JAK1* mutations, mutated tumors also had a modestly lower level of infiltration of cytotoxic lymphocytes (analyzed based on gene expression; *P* = 0.01; Additional file 2: Figure S4g).

### Immune cell infiltration is strongly associated with CMS1, independent of *JAK1* mutation status

To further explore potential correlates to immune cell infiltration, tumors were classified according to the gene expression-based CMS classes of CRC [42]. In Norwegian series I, 44 (60%), 2 (3%), 12 (16%), and 3 (4%) of the MSI+ tumors were classified as CMS1, CMS2, CMS3, and CMS4, respectively (Additional file 2: Figure S5a). Among the exome-sequenced tumors, the mean number of mutations was higher in CMS1 than the other three CMS classes (1964 versus 1537 mutations, respectively; *P* = 0.08 by Welch’s *t*-test; Additional file 2: Figure S5b). This was also found among MSI+ tumors in TCGA (mean mutation load 1499 versus 1097, respectively; *P* = 0.03). There was no enrichment for *JAK1* mutations in any of the CMS classes (*P* = 0.7 by Fisher’s exact test); mutations were found in 11% of CMS1 tumors and 18% of CMS3 tumors in Norwegian series I (the remaining mutations were found in unclassified tumors).

Gene set expression analyses revealed that the distinct biological properties of CMS1 were recapitulated also among MSI+ tumors only (Additional file 2: Figure S5c; Additional file 1: Table S11). In comparison with CMS2–4, tumors in CMS1 had a significantly higher level of infiltration of cytotoxic lymphocytes and a higher immune-score (ESTIMATE; Fig. 4e; Additional file 2: Figure S5d). This was independent of *JAK1* mutation status, and analyzing tumors wild type for *JAK1* only, CMS1 tumors still had a high level of immune cell infiltration (Additional file 2: Figure S5f). Furthermore, CMS1 tumors had strong PD-1 signaling and JAK-STAT signaling (Fig. 4e), independent of *JAK1* mutation status (Additional file 2: Figure S5g).

### *JAK1* loss-of-function mutations and CMS1 are positive prognostic factors in MSI+ CRC

Considering the substantial molecular heterogeneity and associations with immunogenicity detected among MSI+ tumors, the distinct genomic features were investigated for associations with patient outcome.

**Fig. 5:**
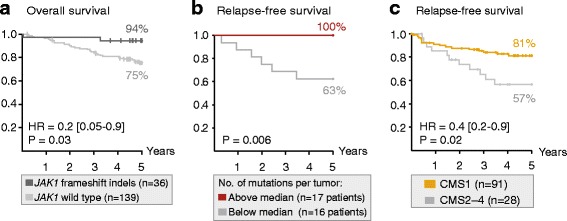
*JAK1* mutations, mutation load, and CMS1 are associated with a good patient outcome. **a** Among 175 patients with MSI+ CRC from Norwegian series I and II and the VICTOR trial, tumors with *JAK1* frameshift indels were associated with a better 5-year overall survival rate (94%) than wild-type tumors (75%; *P* value from Wald’s test). **b** Among the 33 exome-sequenced tumors in Norwegian series I, tumors with a mutation load above the median number of mutations were associated with a better 5-year relapse-free survival rate (100%) than tumors with a low mutation load (63%; *P* value from the log-rank test). **c** Among 119 patients in Norwegian series I and a publicly available dataset (GEO accession number GSE39582), patients in CMS1 had a significantly better 5-year relapse-free survival rate (81%) than patients in CMS2-4 (57%, *P* value from Wald’s test)

Among tumors analyzed for *JAK1* mutations, clinical follow-up data reporting OS were available for 175 of the 182 Norwegian and British patients (Table 1; Fig. 1a). Patients with a *JAK1* frameshift indel (*n* = 36) had an OS rate of 94%, significantly higher than the 75% OS rate of patients wild type for *JAK1* (*n* = 139; hazard ratio (HR) from Cox’s regression 0.2 [95% confidence interval 0.05–0.9], *P* value from Wald’s test of predictive potential 0.03; Fig. 5a). The positive prognostic association of *JAK1* mutations was seen in all the three patient series individually (although not statistically significant; Additional file 2: Figure S6a). In multivariable analysis, the prognostic association was independent of patient age (above versus below median), patient gender, and tumor localization (right versus left and rectum; multivariable HR = 0.2 [0.06–1.0], *P* = 0.05). Furthermore, the majority of the patients (89%; 155 of 175) were diagnosed with stage II or III MSI+ CRC, and the prognostic association of *JAK1* frameshift indels was independent of cancer stage in these patients (multivariable HR = 0.1 [0.02–0.8], *P* = 0.03; Additional file 2: Figure S6b).

Although there was no difference in the mutation load between tumors with and without *JAK1* frameshift indels (*n* = 33 patients in Norwegian series I), a large burden of small exonic mutations (above the median; 1676 mutations) was also associated with a favorable patient outcome (Fig. 5b), independent of cancer stage (Additional file 2: Figure S6c). In contrast, neither the MSI score nor the clonal composition of the tumors (analyzed as the number of subclones predicted by either SciClone or EXPANDS) were associated with patient survival.

The strong association between CMS1 and immune cell infiltration suggests that CMS1 is also a favorable prognostic factor in MSI+ CRC. Indeed, among 119 patients (with known CMS class and clinical information) from Norwegian series I and a publicly available dataset (GSE39582) [45], patients with CMS1 tumors (*n* = 91) had a 5-year RFS rate of 81%, significantly higher than the survival rate of 57% for patients with CMS2–4 tumors (*n* = 28) (HR = 0.4 [0.2–0.9], *P* = 0.02; Fig. 5c). A similar prognostic association was found for 5-year OS (HR = 0.5 [0.2–1.0], *P* = 0.06; Additional file 2: Figure S6d). CMS1 was associated with a better outcome than each of the subtypes CMS2, CMS3, and CMS4 separately (Additional file 2: Figure S6e). In a multivariable model including patient age, gender, and cancer stage, CMS1 (versus CMS2–4) was an independent prognostic factor (multivariable HR = 0.4 [0.2–0.9], *P* = 0.02; Additional file 1: Table S12).

Combined survival analysis of CMS1 and *JAK1* mutations was possible for 51 patients in Norwegian series I. Here, *JAK1* mutations provided additional prognostic information to CMS1. Of note, the sample numbers in the individual subgroups were low (four and two *JAK1* mutated tumors in CMS1 and CMS2–4, respectively; Additional file 2: Figure S6f). However, in a multivariable model, the prognostic value of CMS1 (versus CMS2–4) was statistically independent of *JAK1* mutations (multivariable HR = 0.2 [0.09–0.7] and 0.3 [0.1–0.9] for 5-year RFS and OS, respectively; *P* = 0.007 and 0.03).

## Discussion

Accurate genome-wide analysis of MSI-type indel mutations has been challenging. Even in the most comprehensive mutational characterization studies of MSI+ CRCs published to date, scoring of indels has been limited either to known homopolymers in selected genes [16, 20, 21] or targeted sequence alignments of a reference set of microsatellite repeats [77], or mutation calling was limited to SNVs [78]. By multilevel genomics and computational analyses, we have identified heterogeneity with clinical relevance within this distinct subtype of colorectal tumors. By exome sequencing, we identified novel, frequent frameshift mutations in four cancer-critical genes (*CRTC1*, *BCL9*, *JAK1*, and *PTCH1*). The majority of mutations in *CRTC1* were subclonal, possibly explaining why these have previously gone unnoticed, and highlighting the importance of high sequencing coverage. Truncating indels in *JAK1*, leading to loss of the entire kinase domain, were found in 20% of a total of 248 tumors. In contrast, *JAK1* indels have previously been reported either as infrequent events in CRC [74] or as specific to MSI+ tumors from the endometrium [77]. We detected frameshift mutations in 18% of MSI+ CRCs from the dataset analyzed in the latter study (TCGA), illustrating the challenge of large-scale analysis of MSI-type mutations.

Consistent with a high level of intra-tumor heterogeneity and the “Big Bang model” of CRC development [79], clonality modeling predicted several subclones in most of the MSI+ tumors. There was no association between tumor clonality and patient survival, which is in contrast to reports from several other cancer types [80, 81]. On the other hand, a favorable outcome was found among patients with a high mutation load. As expected, the mutation load was strongly correlated to the predicted neoantigen load, and the level of immune cell infiltration was significantly higher in MSI+ compared to MSS tumors, consistent with recent results [37]. However, within the subgroup of MSI+ tumors, we found no association between the mutation load, or predicted neoantigen load, and the level of immune cell infiltration. This may be explained by technical issues, including the low sample number and insufficient statistical power, lower fidelity of mutation detection among hypermutated tumors, and lower sensitivity of immune cell infiltration detection based on gene expression compared to immunohistochemistry. However, similar and seemingly disparate results have recently been described in melanoma. Here, the mutation load of pre-treatment biopsies was associated with improved patient survival, but not with response to anti-PD-1 treatment, which in turn is associated with intra-tumor T-cell infiltration [40], suggesting additional determinants of tumor immunogenicity. We found immune cell infiltration to be highly dependent on the CMS class of the tumors. CMS1 is an immunogenic subtype of CRC, associated with a high prevalence of MSI [42, 43], and this study is, to our knowledge, the first to describe CMS1 as a particularly immunogenic subtype also specifically among MSI+ tumors. Clinical relevance was reinforced by a strong association between CMS1 and patient survival. CMS1 was originally described as a poor prognostic factor after relapse [42]; however, among patients with MSI+ CRC, we identify CMS1 as a favorable prognostic factor, independent of important clinicopathological parameters.

Positive prognostic associations were observed also for truncating *JAK1* mutations, which is in contrast to recent results describing a poor outcome in patients with skin subcutaneous melanoma, breast invasive carcinoma, and prostate adenocarcinoma (no prognostic associations were detected in CRC in this study) [73]. These are cancer types with a low prevalence of MSI [15], suggesting heterogeneous prognostic associations according to the mutation phenotype (MSI status). JAK-STAT signaling regulates diverse cellular processes and may have oncogenic potential [82]. Preclinical data suggest that inhibition of JAK1 can block colorectal tumor growth [83], supporting a potential positive prognostic value of inactivating mutations, but additional analyses are required to explain the role of *JAK1* mutations in the progression of MSI+ CRC. Furthermore, multivariable analyses indicate that *JAK1* mutations and CMS1 have independent prognostic value, but combined analyses in larger patient series are needed. Still, the promising results observed for *JAK1* mutations and CMS1 in altogether four independent patient series reinforce the potential for molecular prognostic stratification of MSI+ CRC.

Although hypermutated tumors generally respond well to anti-PD-1 treatment, resistance is commonly observed in metastatic MSI+ CRC. Homozygous *JAK1* loss-of-function mutations, resulting in an inability to signal in the IFN-γ response pathway and with subsequent loss of PD-L1 expression, has been identified as one potential resistance mechanism [73]. At the gene expression-level, we observed similar associations with *JAK1* mutations in our series of primary MSI+ CRCs, including up-regulation of transcriptional signatures associated with innate resistance to anti-PD-1 treatment in melanoma [40]. However, complete loss of JAK1 function is likely required to confer treatment resistance [73], and clonality modeling suggested that the mutations were heterozygous. We speculate that primary tumors with heterozygous, truncating mutations are more prone to biallelic inactivation and to development of resistance in the metastatic setting. However, the need for a second, inactivating hit suggests that *JAK1* mutations do not account for the majority of cases with anti-PD-1 resistance. This is supported by the favorable outcome among patients with mutated tumors, predicting a low mutation frequency in metastatic disease. Consequently, there is still a need to uncover additional mechanisms of resistance to anti-PD-1 treatment in metastatic MSI+ CRC.

## Conclusions

We have identified novel frequent frameshift mutations and molecular heterogeneity with clinical relevance in primary MSI+ CRC. A high mutation load was found to correlate with the predicted neoantigen load, but immune cell infiltration was highest in the transcriptomic CMS1 subgroup, and CMS1 was a predictor of a good patient outcome. Of particular interest, considering the potential for prediction of resistance to anti-PD-1 treatment, was the validation of a high prevalence of truncating *JAK1* indels in independent patient series. However, the mutations were associated with a favorable patient prognosis, suggesting a low mutation frequency in metastatic disease, and reinforcing the need to discover additional mechanisms of resistance to immunotherapy.

## Additional files

Additional file 1: Supplementary Tables. An Excel file containing Supplementary Tables S1-S12. (XLSX 8497 kb)

Additional file 2: Supplementary Text and Figures. A Word file containing Supplementary Text and Figures S1-S6. (DOCX 1054 kb)

## Abbreviations

BAM: Binary Alignment Map
CGC: Cancer Gene Census
CMS: Consensus molecular subtype
CNA: Copy number aberration
CRC: Colorectal cancer
EMT: Epithelial-to-mesenchymal transition
GEO: Gene Expression Omnibus
HR: Hazard ratio
indel: Insertions and/or deletions
LOH: Loss of heterozygosity
MMR: DNA mismatch repair
MSI: Microsatellite instability
MSS: Microsatellite stability
OS: Overall survival
RFS: Relapse-free survival
RMA: Robust multi-array average
SAM: Sequence Alignment Map
SNV: Single nucleotide variant
TCGA: The Cancer Genome Atlas
VAF: Variant allele frequency.
