# Supplementary material for: Multilevel genomics of colorectal cancers with microsatellite instability—clinical impact of JAK1 mutations and consensus molecular subtype 1
Source: Genome Med. 2017 May 24;9:46. doi: 10.1186/s13073-017-0434-0 (PMC5442873; doi:10.1186/s13073-017-0434-0)
Supplement: Supplementary file 2 — Supplementary Text and Figures. A Word file containing Supplementary Text and Figures S1-S6. (DOCX 1054 kb) [file 13073_2017_434_MOESM2_ESM.docx]

# Additional file 2: Supplementary Text and Figures

## Comparison of SciClone and EXPANDS for tumor clonality modeling

Tumor clonality was modeled for 27 tumors based on two different algorithms, SciClone and EXPANDS. In general, EXPANDS predicted more subclones per tumor than SciClone (median 3, range 1 to 8 *versus* median 2, range 1 to 3, respectively); however, the predicted number of subclones and the predicted cancer cell fraction (determined from the subclone with the largest cellular prevalence) from the two algorithms were proportional among the tumors (Spearman correlations 0.5 and 0.7, P = 0.02 and 7×10^-6^, respectively). Furthermore, both algorithms predicted multiple subclones in the majority of tumors (all tumors except three [89%] and one [96%], respectively, for SciClone and EXPANDS). Based on EXPANDS, the number of predicted subclones were independent of the number of mutations (Spearman correlation 0.3, P = 0.2), and the amount of truncal mutations between the full exome and genes in the Cancer Gene Census was proportional (Spearman correlation 0.9, P = 7×10^-13^), confirming results by SciClone.


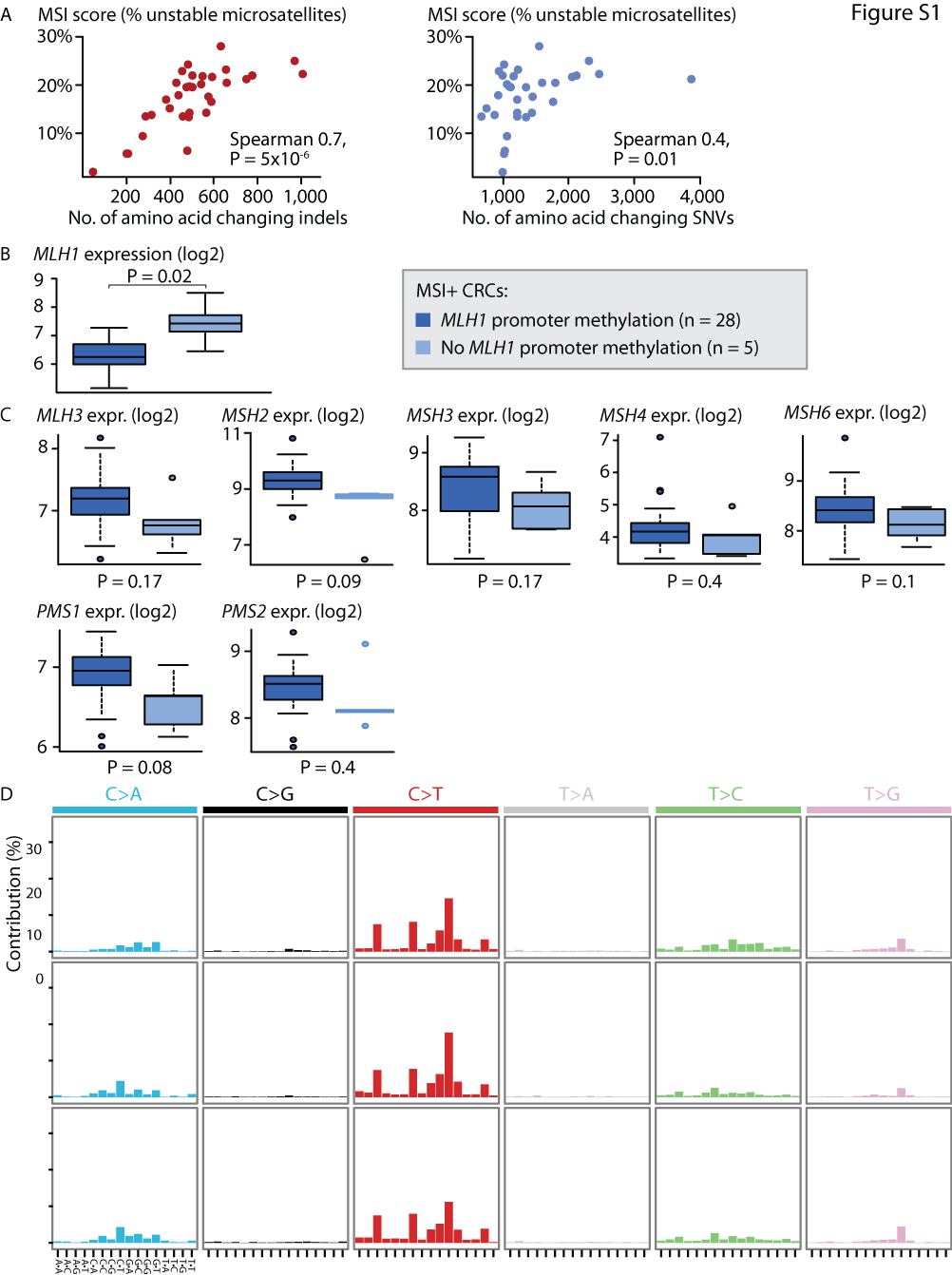


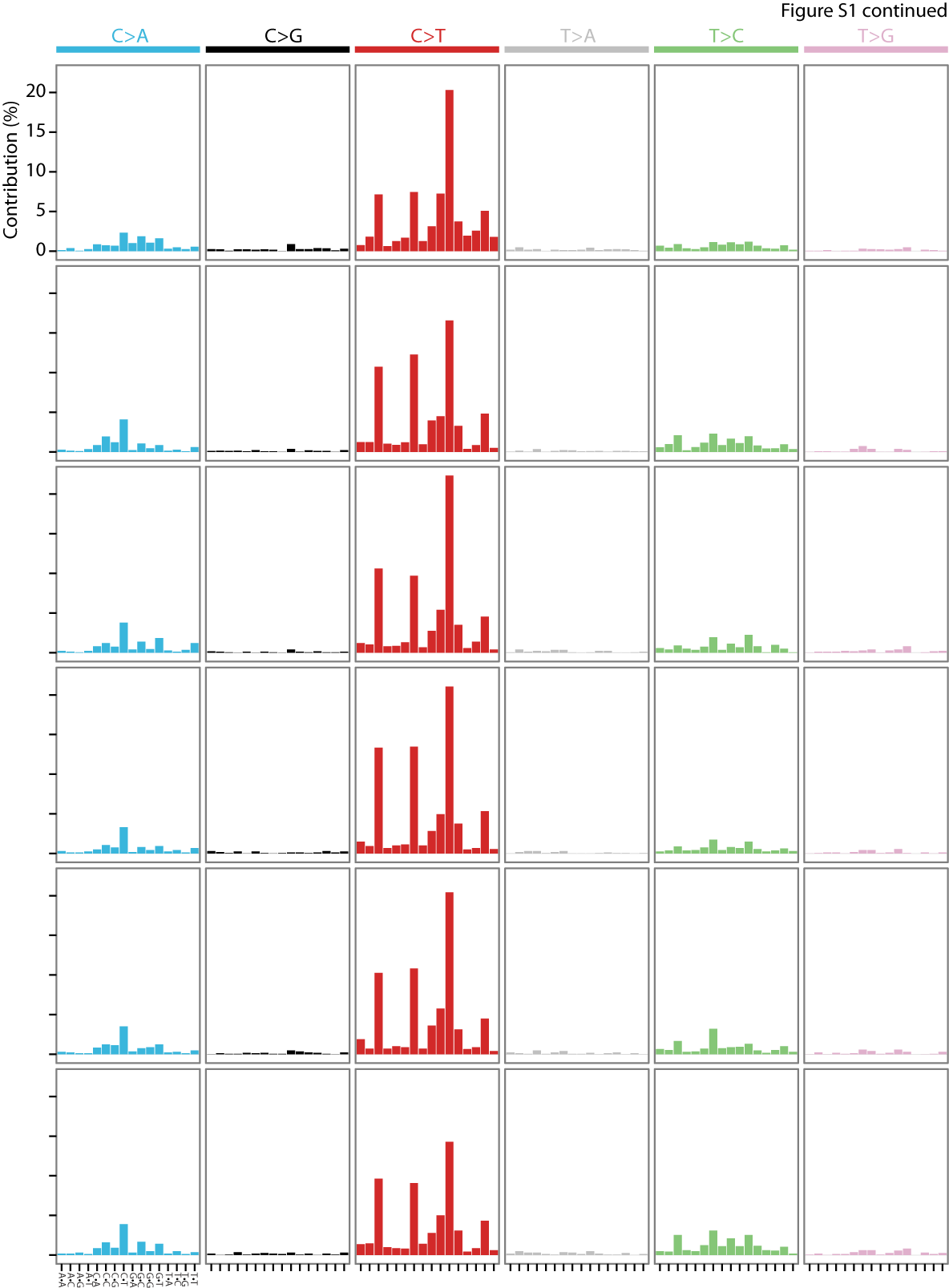

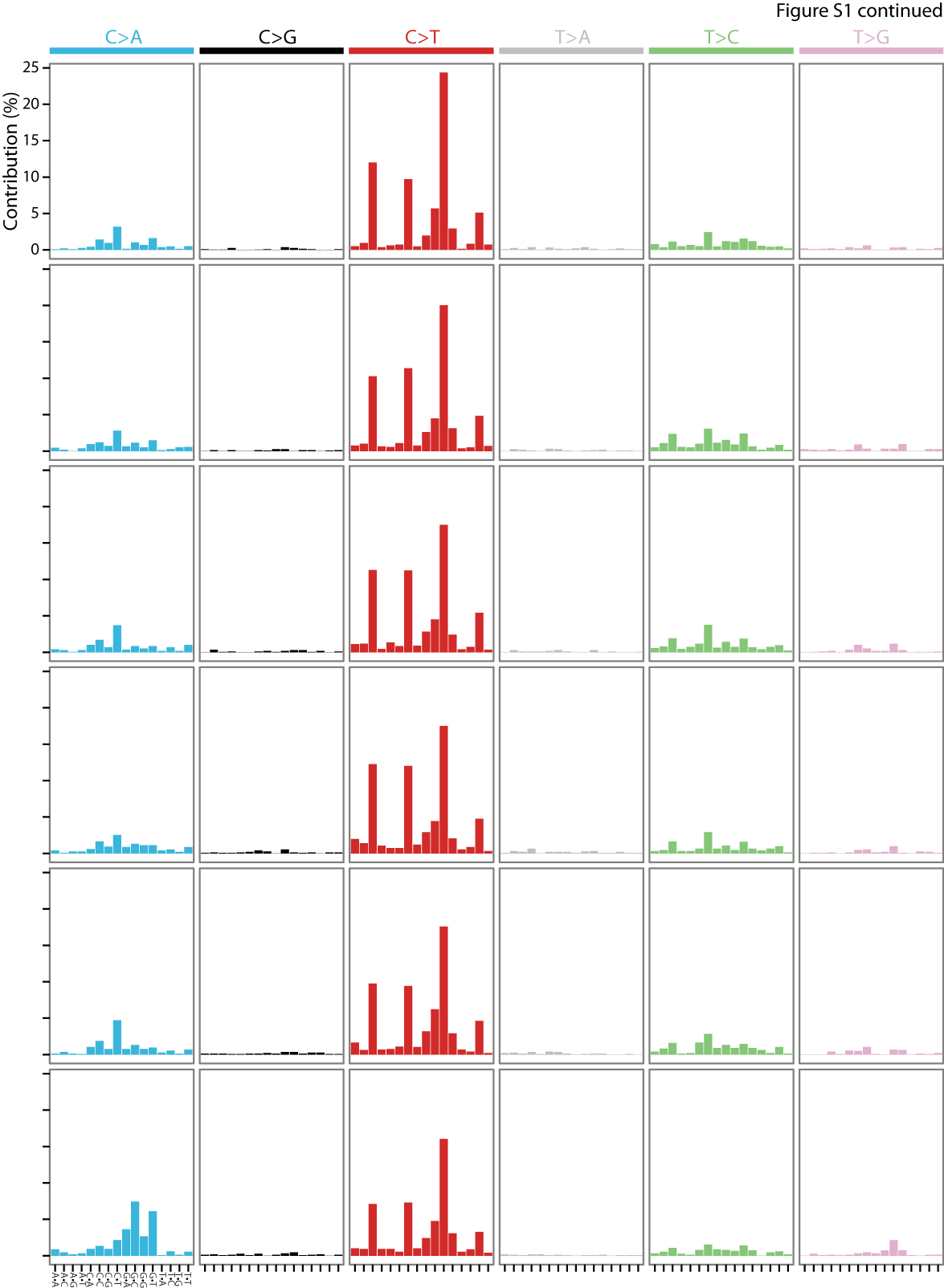

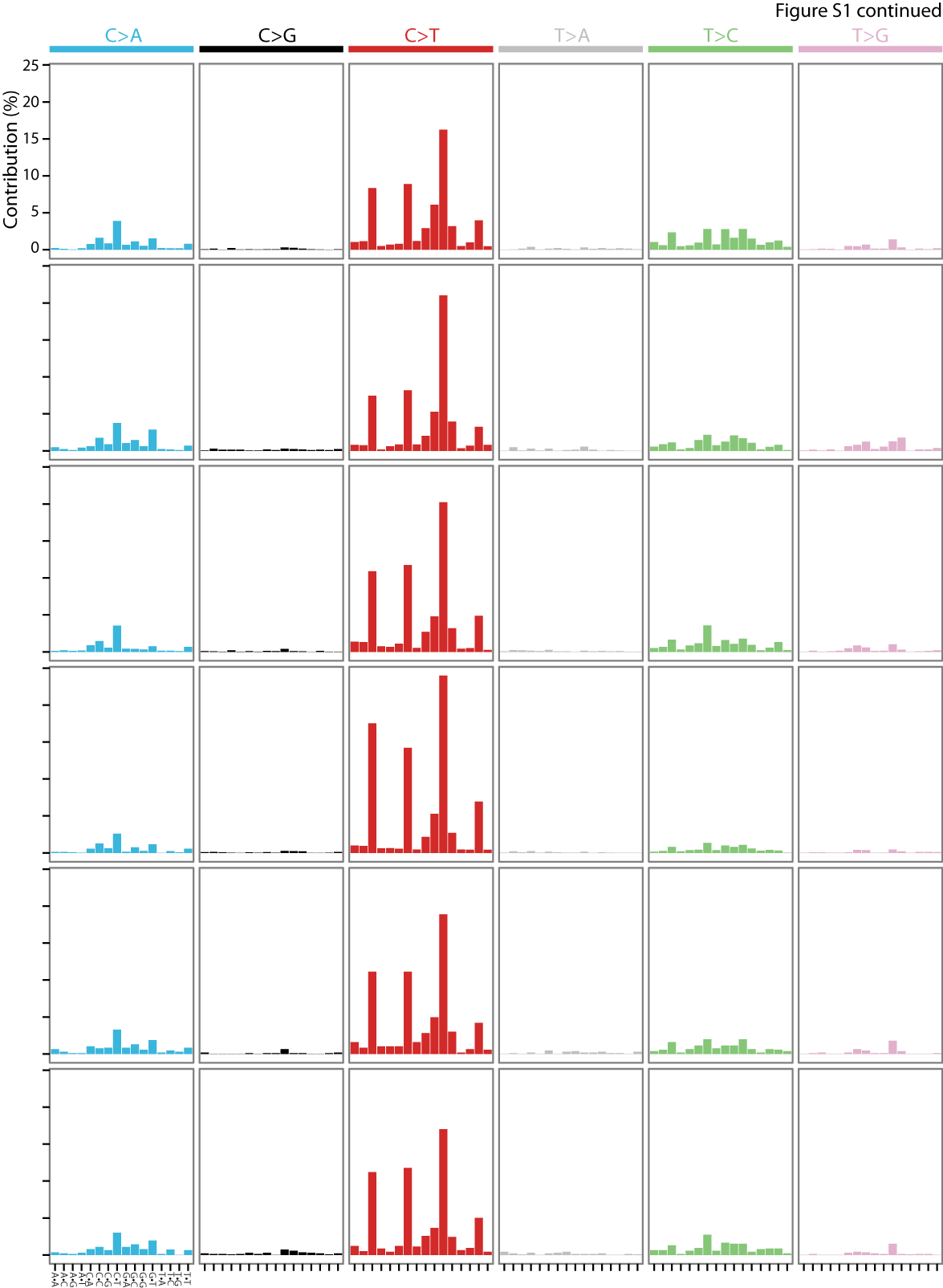

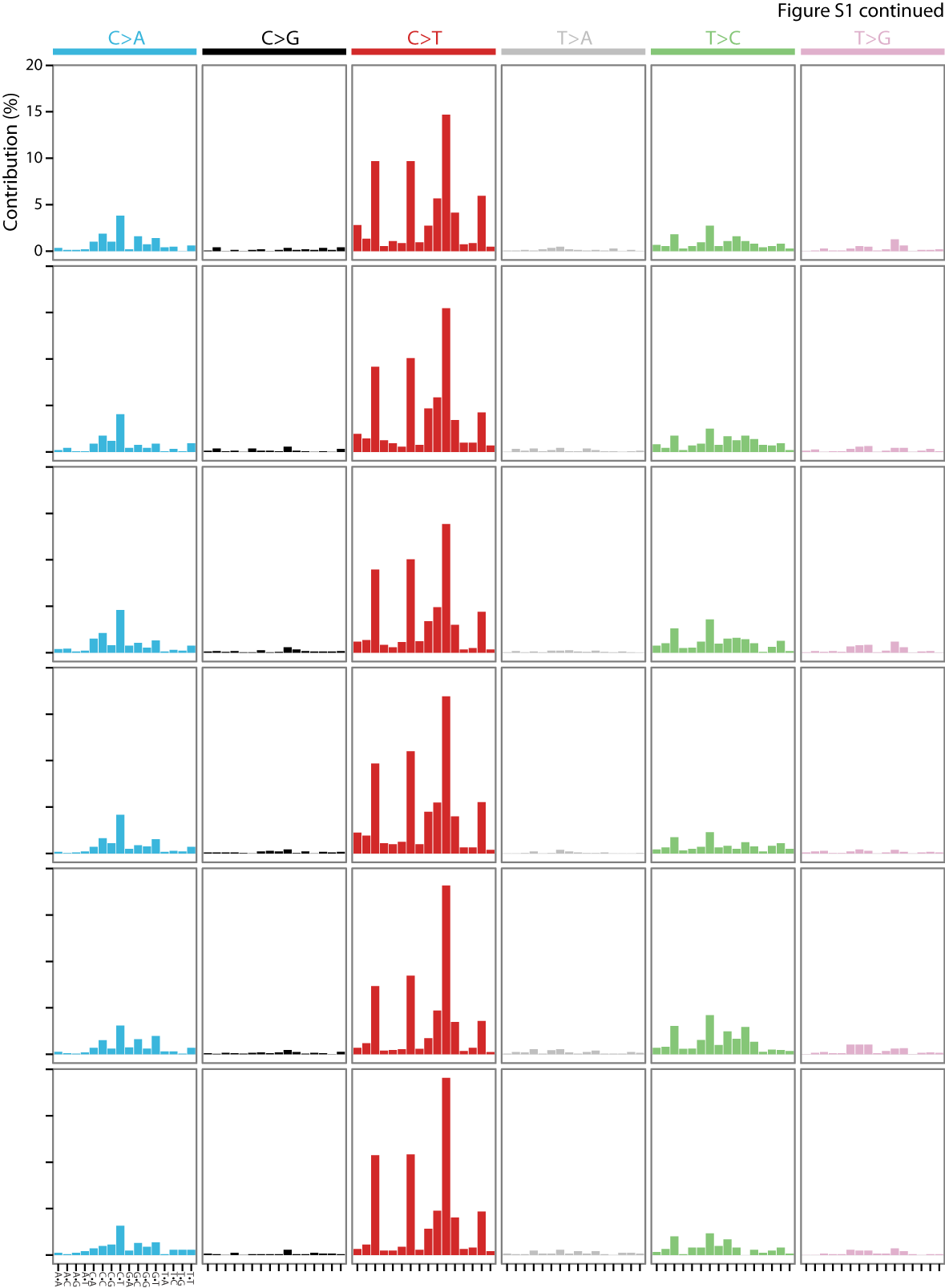

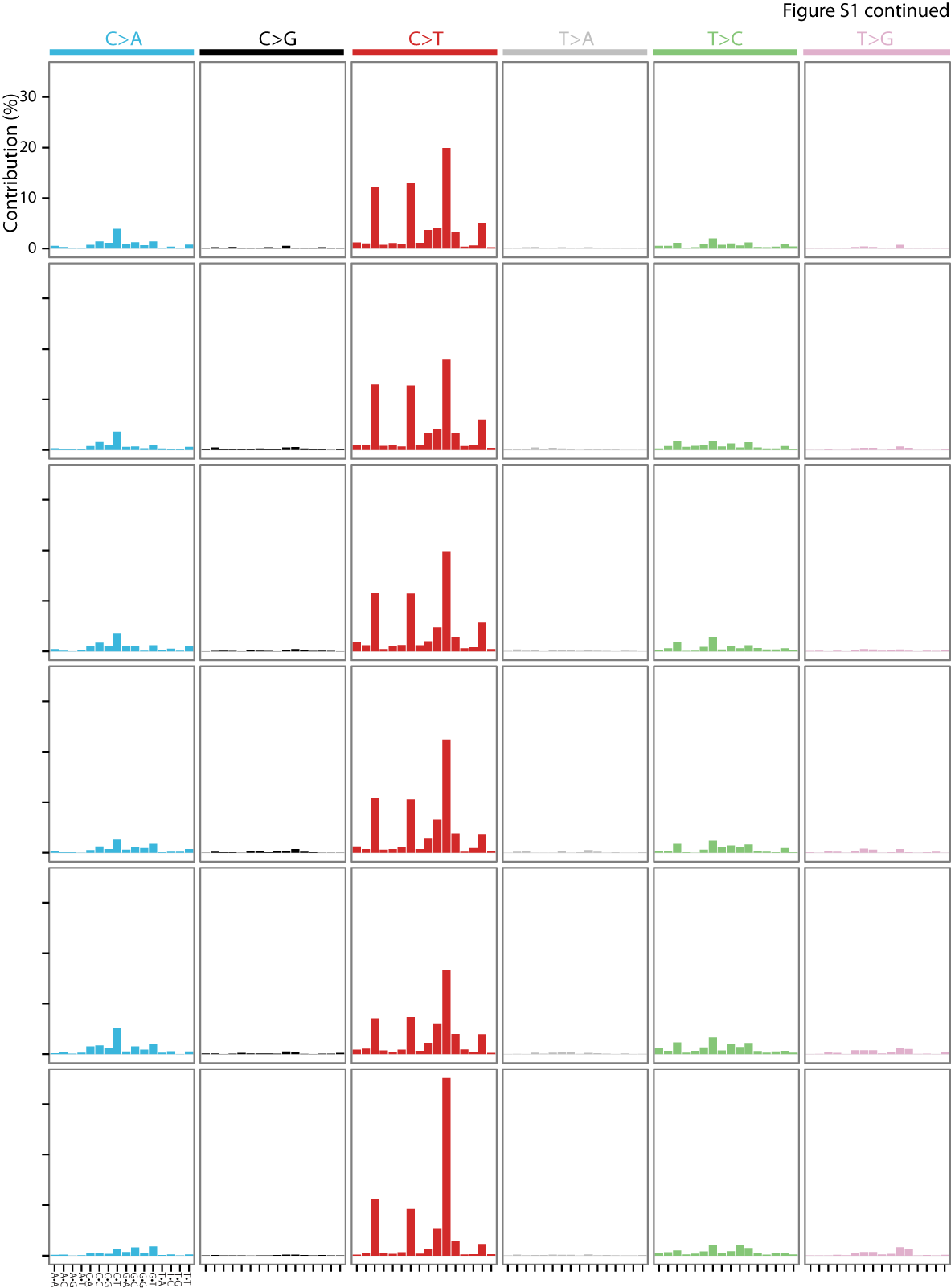

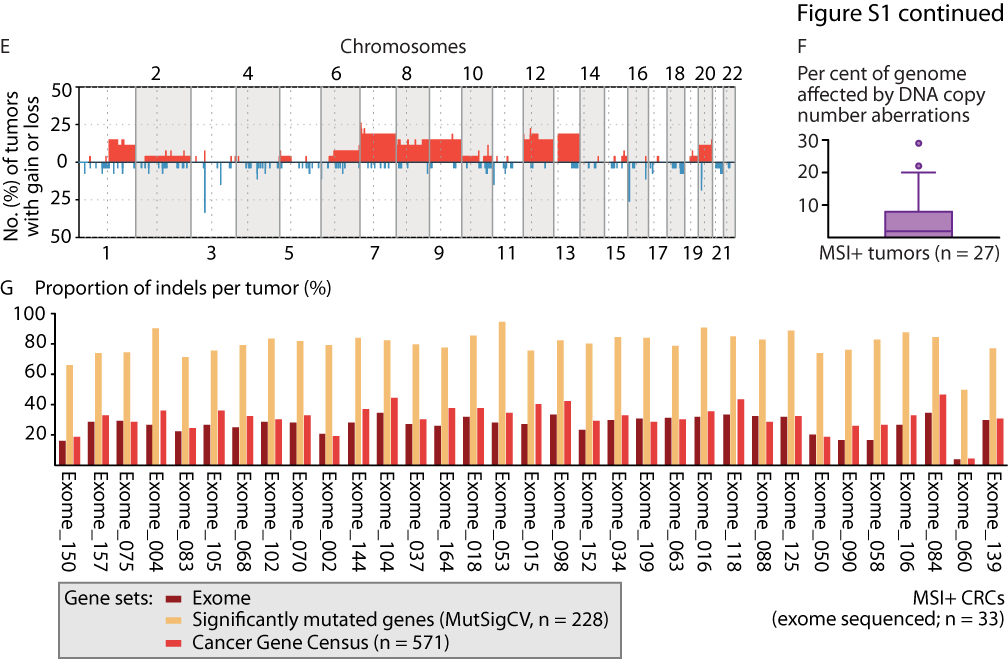


## Figure S1. MSI score, expression of mismatch repair genes, exome mutations, and DNA copy number aberrations in the 33 exome sequenced MSI+ CRCs in Norwegian series I

A) The MSI score, representing the percentage of somatically unstable microsatellites per tumor (% of microsatellites with indels, calculated using the algorithm MSIsensor) was strongly correlated to the number of indels per tumor, but only weakly associated with the number of SNVs. B) Twenty-eight of the 33 tumors had *MLH1* promoter methylation and a significantly lower *MLH1* gene expression than the five tumors without methylation (P = 0.02 from Welch’s two-sample t-test with unequal variances). C) In contrast, the other seven mismatch repair genes had reduced expression levels in the tumors without *MLH1* promoter methylation, although not statistically significant (P-values from Welch’s t-test are indicated below each plot). The color code is the same as in part B. D) The frequency of different types of substitutions per tumor (each row represents one tumor). The vertical axes represent the proportion of substitutions in each of six categories (indicated in the top panel), classified according to the sequence context (the flanking nucleotides are indicated on the horizontal axis; the axis notation is the same for all six substitution categories). All the 33 tumors had a similar mutation distribution, corresponding to mutational signature 6 as designated in COSMIC, which is associated with a defective DNA mismatch repair-machinery. E) The MSI+ tumors had few DNA copy number aberrations (CNAs). Twenty-seven of the 33 exome sequenced tumors were analyzed for CNAs and aberration frequencies of gains (red) and losses (blue) along the genome are shown on the vertical axis (chromosome numbers are indicated on the horizontal axes; chromosomes and chromosome arms are separated by vertical lines). Recurrent, but relatively low-frequency CNAs (26% or fewer of the patients) included gain of whole chromosomes 7, 8, 9, 12 and 20 and chromosome arms 1q and 13q, as well as a focal gain at 10q25.3, including *NRAP*. Recurrent copy number losses included focal deletions at 3p14.2, 16p13.3 and 20p12.1, including *FHIT*, *RBFOX1*, and *MACROD2* (loss in 33%, 26% and 19% of the patients, respectively). F) The median proportion of the genome affected by CNAs across the 27 tumors was 2% (range 0 to 29%). The most heavily affected tumors (five tumors with more than 10% of the genome affected) had mostly large aberrations affecting whole chromosomes or chromosome arms, *i*.*e*., only a small number of events. G) The proportion of the total number of mutations that are frameshift indels per tumor are plotted separately for the full exome, genes identified by MutSigCV as significantly mutated, and genes in the Cancer Gene Census, as indicated. There is enrichment for indels both in the significantly mutated genes and the genes in the Cancer Gene Census, compared with the full exome. In this plot, the 33 tumors are arranged in the same order as in Figures 2a-c, *i*.*e*. according to a decreasing total number of amino acid changing mutations.


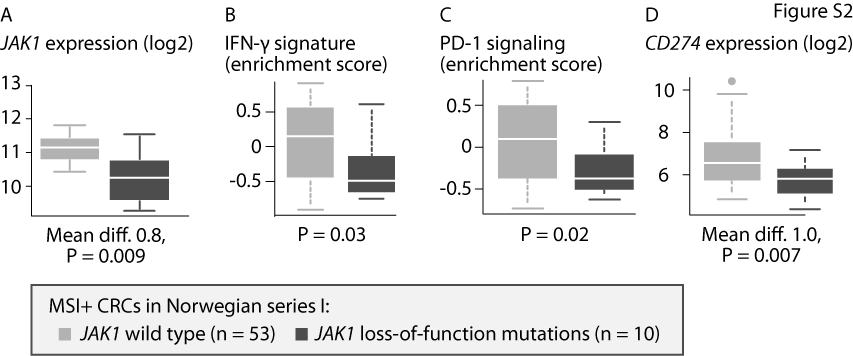


## Figure S2. Transcriptional consequences of *JAK1* loss-of-function mutations in MSI+ CRCs

In Norwegian series I, tumors with *JAK1* loss-of-function mutations had down-regulation of A) *JAK1* gene expression, B) a 6-gene IFN-γ expression signature, C) PD-1 signaling, and D) the gene encoding the PD-L1 ligand, *CD274*. For the IFN-γ (Chow *et* *al*., J Clin Oncol. 2016;34:suppl; abstr 6010) and PD-1 signaling (Reactome Pathway Database; identifier R-HSA-389948) gene expression signatures, sample-wise gene set enrichment scores were calculated using the R package GSVA. P-values were calculated by Welch’s two sample t-tests.


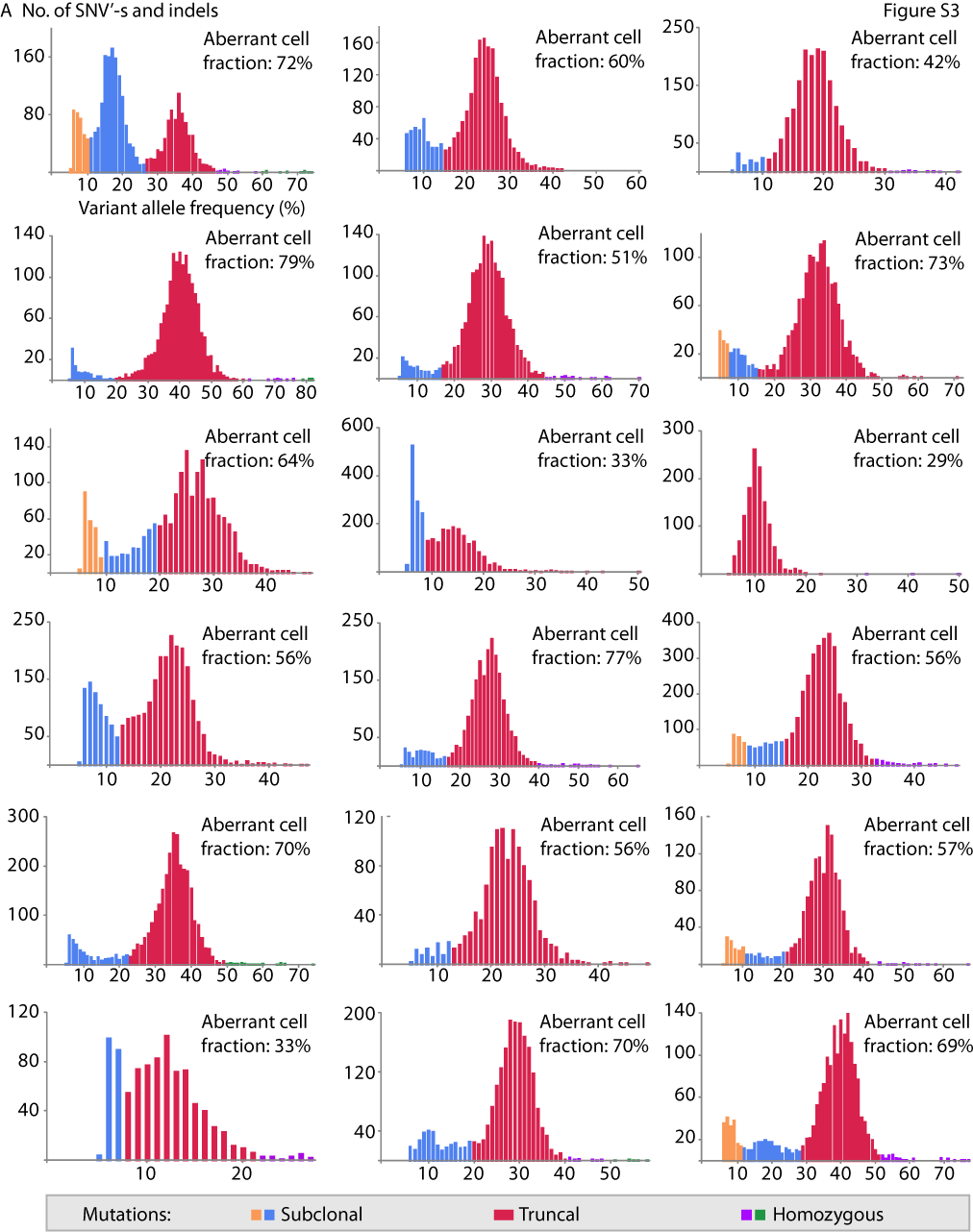


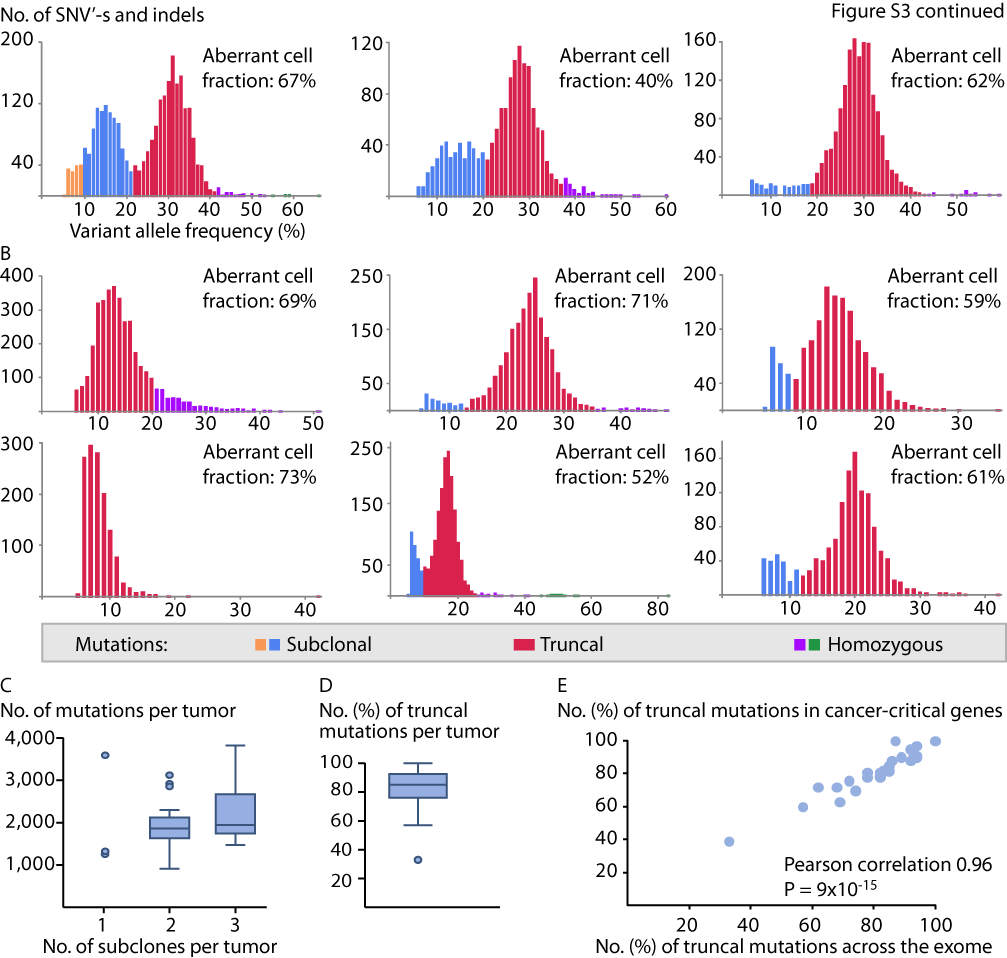


## Figure S3. Clonality of MSI+ CRCs and exonic mutations

A) The clonal composition of 27 MSI+ CRCs was modeled from small, exonic mutations unaffected by DNA copy number aberrations. Each plot represents one sample, and the different colors indicate separate mutation clusters, as estimated from the variant allele frequencies (VAFs) of the mutations. In tumors (n = 21) with a mutation cluster with a mean VAF corresponding to approximately 50% of the estimated aberrant cell fraction, heterozygous truncal mutations (red) can unambiguously be scored, whereas B) in tumors (n = 6) with a discrepancy between the mean VAFs of the mutation clusters and the estimated aberrant cell fraction, the determination of a truncal mutation cluster is less certain. The number of clones per tumor was summarized from distinct mutation clusters of truncal and subclonal mutations. All tumors except three had multiple mutation clusters (subclones). The samples are sorted in the same order as in Table S10 in Additional file 1. C) Among the 27 tumors, the number of subclones per tumor (ranging from one to three) was not associated with the total number of mutations. D) The majority of mutations were truncal in all except one of the 27 tumors; the proportion of truncal mutations per tumor ranged from 33% to 100% (median 85%). E) The proportion of mutations in cancer-critical genes (among the 571 genes in the Cancer Gene Census) that were truncal was strongly correlated with the corresponding proportion among all the genes in the exome.


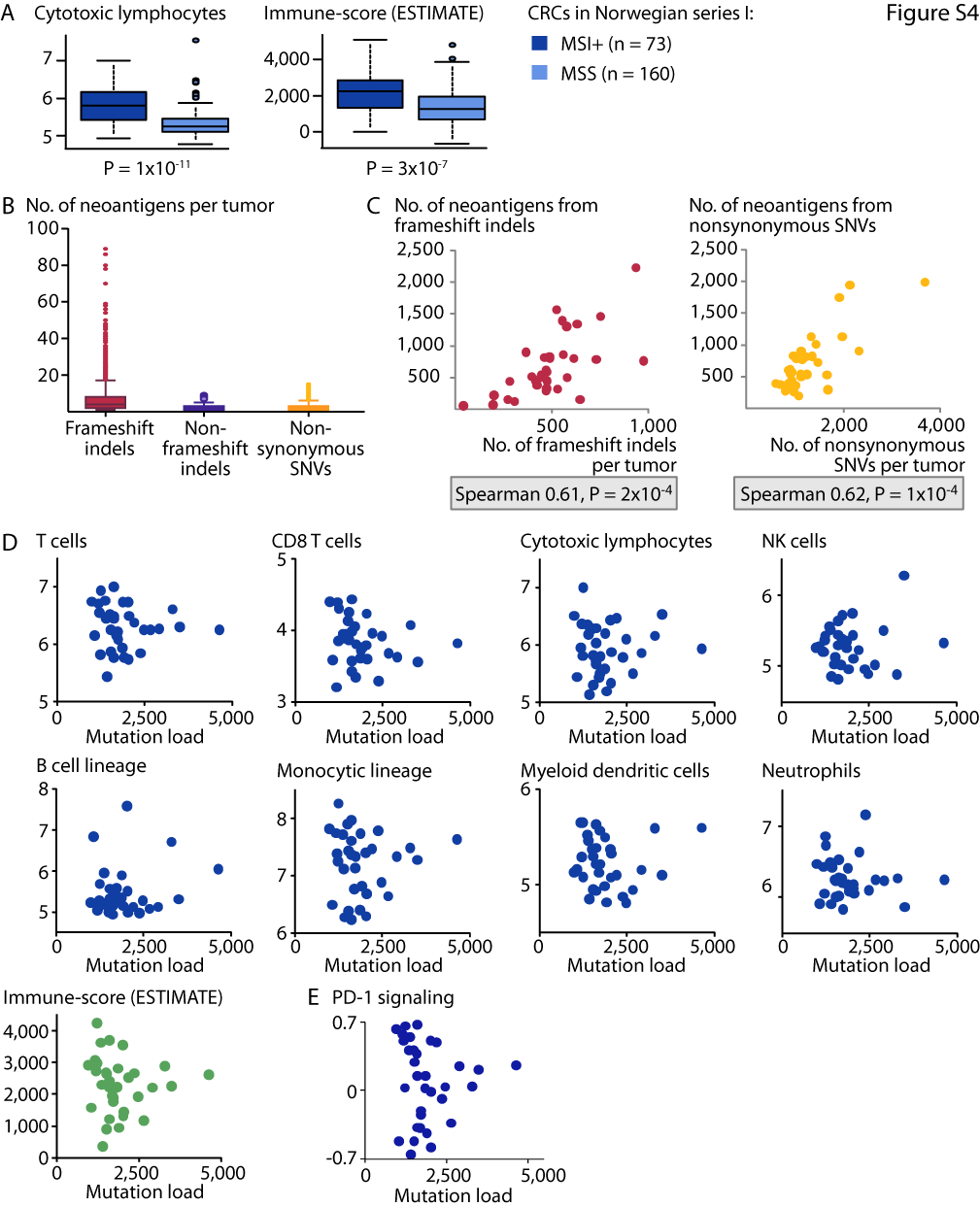


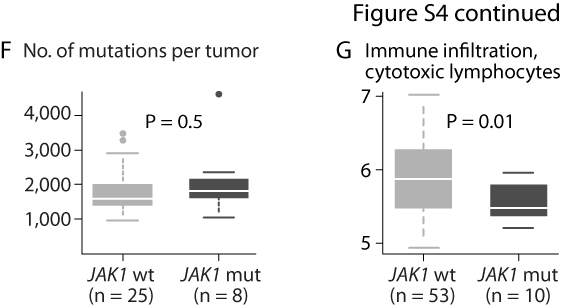


## Figure S4. Mutation load, predicted neoantigens, *JAK1* mutations and immune cell infiltration in MSI+ CRCs in Norwegian series I

The level of infiltration of immune cells in the tumors was evaluated based on gene expression using the R packages MCPcounter and ESTIMATE. A) Compared with tumors with microsatellite stability (MSS), MSI+ tumors had a significantly higher level both of infiltration of cytotoxic lymphocytes and the immune-score ESTIMATE (evaluated by Welch’s t-test). B) In the 33 exome sequenced tumors, neoantigens were predicted from somatic mutations in highly expressed genes. In particular frameshift indels were predicted to create a large number of neoantigens in individual tumors, and all mutations creating more than 15 neoantigens in one tumor were frameshift indels. C) The numbers of neoantigens predicted both from frameshift indels (red) and nonsynonymous SNVs (yellow) were correlated with the corresponding numbers of mutations per tumor. D) There was no association between immune cell infiltration and the number of amino acid-changing mutations (mutation load) per tumor. The Pearson correlations ranged from -0.3 to 0.01 (P > 0.1). Analyzing the associations between immune infiltration and the predicted neoantigen load per tumor, the Pearson correlations ranged from -0.3 to 0.06 (P > 0.08). E) Furthermore, the mutation load was not associated with PD-1 signaling (evaluated by sample-wise gene set expression enrichment analysis using the R package GSVA; Spearman’s correlation -0.2, P = 0.2). F) Tumors with *JAK1* frameshift mutations did not have a significantly different mutation load compared with wild type tumors. G) However, mutated tumors had a significantly lower level of infiltration of cytotoxic lymphocytes.


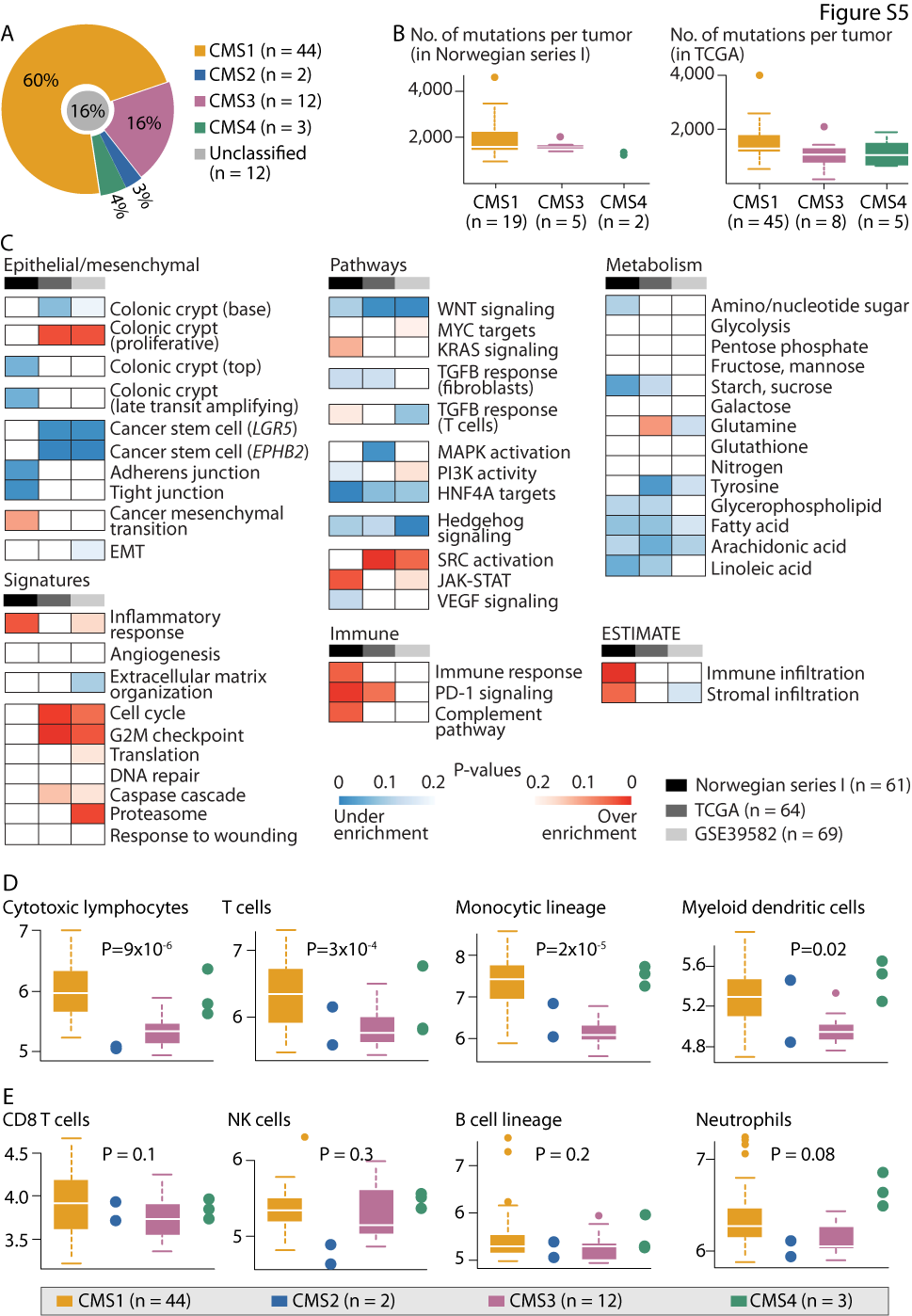


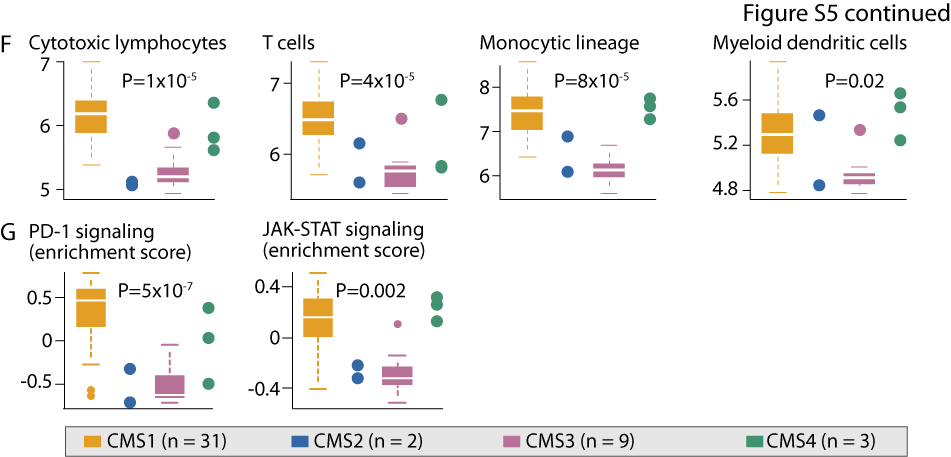


## Figure S5. MSI+ CRCs in CMS1 have a higher mutation load and level of immune cell infiltration than MSI+ tumors in CMS2-4, independent of *JAK1* mutation status

A) Among the 73 MSI+ tumors in Norwegian series I with available gene expression data, 61 tumors were confidently assigned a CMS-subtype and twelve were not classified. As expected, the majority (60%) of the tumors was classified as CMS1, and 16% were assigned to the CMS3-subtype. B) MSI+ tumors in CMS1 had a higher mutation load than tumors in the other CMS classes, both in Norwegian series I (among the 26 of the 33 exome sequenced tumors that were confidently assigned to a CMS class) and TCGA, although the difference was statistically significant in the TCGA dataset only (P = 0.08 and 0.03, respectively, by Welch’s t-test). C) Gene set expression analyses comparing MSI+ tumors in CMS1 *versus* CMS2-4 in Norwegian series I (black), TCGA (dark grey) and GSE39582 (light grey). Red and blue boxes indicate unadjusted P-values for positive and negative enrichment, respectively, in CMS1 for each of a customized collection of CRC relevant gene sets (overview in Additional file 1: Table S11). In Norwegian series I, the distinct biological properties of CMS1 were recapitulated also among the MSI+ tumors. MSI+ tumors in CMS1 had high expression of genes involved in immune responses, immune evasion (PD-1 signaling), inflammatory response, and JAK-STAT signaling, and had mesenchymal characteristics in comparison with the other CMS classes. The same associations were not seen as clearly in the other two patient series; however, WNT and hedgehog signaling, as well as HNF4α targets and metabolic processes were negatively enriched in CMSI in all three patient series. In comparison with tumors in CMS2-4, tumors in CMS1 had a significantly higher level of infiltration of several types of immune cells, indicated in part D), but not of the cell types indicated in part E). When analyzing only tumors wild type for *JAK1*, CMS1 tumors still had a significantly higher level of F) immune cell infiltration and G) PD-1 and JAK-STAT signaling than CMS2-4 tumors. P-values were calculated by comparing CMS1 *versus* CMS2-4 tumors by Welch’s two-sample t-tests.


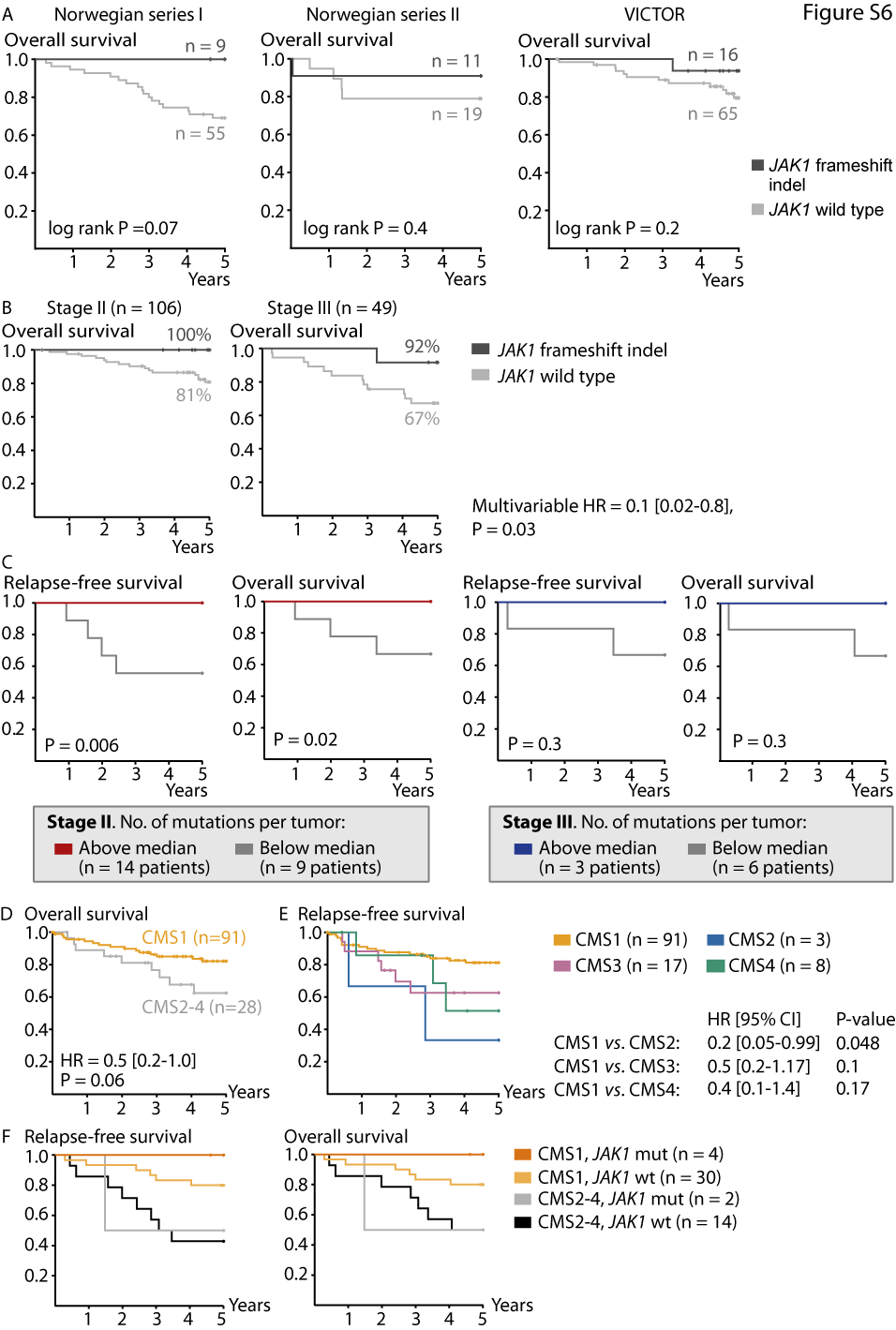


## Figure S6. *JAK1* loss-of-function mutations, mutation load and CMS1 are associated with a favorable patient outcome in MSI+ CRC

A) Among 175 patients from Norwegian series I and II and the VICTOR trial with *JAK1* mutation status and clinical follow-up data available, *JAK1* mutations were associated with a favorable 5-year overall survival rate compared to wild type tumors in each patient series separately, although not statistically significant. B) Among the 155 patients diagnosed with stage II or III MSI+ CRC (89% of the totally 175 patients), *JAK1* frameshift indels were associated with a favorable patient outcome within both stages. The hazard ratio, HR, at five years was calculated by multivariable Cox’s regression including *JAK1* mutation status and cancer stage. The P-value was calculated by Wald’s test of predictive potential. C) In Norwegian series I (n = 33 patients with exome-sequenced MSI+ tumors), tumors with a mutation load above the median number of mutations per tumor (1,676 mutations) were associated with a good relapse-free and overall survival rate in comparison with tumors with a low mutation load, both in patients with stage II and stage III cancer individually. In stage III, the association was not statistically significant due to the small number of patients. D) Among 119 patients with MSI+ CRC in Norwegian series I and GSE39582, CMS1 was associated with a better 5-year OS rate than CMS2-4 (although with borderline statistical significance). E) Furthermore, CMS1 was associated with a better 5-year RFS rate than each of the subtypes CMS2, CMS3 and CMS4 separately, although not statistically significance due to the limited number of samples in CMS2-4. F) Among the 51 patients in Norwegian series I confidently assigned to a CMS class and with *JAK1* mutation status available, *JAK1* mutation status provided additional prognostic information to CMS1.
